# Accurate phenotype-to-genotype mapping of high-diversity yeast libraries by heat-shock-electroporation (HEEL)

**DOI:** 10.1128/mbio.03197-24

**Published:** 2024-12-20

**Authors:** Marcus Wäneskog, Emma Elise Hoch-Schneider, Shilpa Garg, Christian Kronborg Cantalapiedra, Elena Schäfer, Michael Krogh Jensen, Emil Damgaard Jensen

**Affiliations:** 1 The Novo Nordisk Foundation Center for Biosustainability, Technical University of Denmark, Lyngby, Denmark; Harvard Medical School, Boston, Massachusetts, USA

**Keywords:** DNA transformation, DNA electroporation, automated genotyping, heat-shock, barcodes, yeast, plasmids, DNA libraries

## Abstract

High-throughput DNA transformation techniques are invaluable when generating high-diversity mutant libraries, a cornerstone of successful protein engineering. However, transformation efficiencies have a direct correlation with the probability of introducing multiple DNA molecules into each cell, although reliable library screenings require cells that contain a single unique genotype. Thus, transformation methods that yield a high multiplicity of transformations are unsuitable for high-diversity library screenings. Here, we describe an innovative yeast library transformation method that is both simple and highly efficient. Our dual heat-shock and electroporation approach (HEEL) creates high-quality DNA libraries by increasing the fraction of mono-transformed yeast cells from 20% to over 70% of all transformed cells, thus allowing for near-perfect phenotype-to-genotype associations. HEEL also allows more than 10^7^ yeast cells per reaction to be transformed with a circular plasmid molecule, which corresponds to an almost 100-fold improvement compared with current yeast transformation methods. To further refine our library screening approach, we integrated an automated yeast genotyping workflow with a dual-barcode design that employs both a single nucleotide polymorphism and a high-diversity region. This design allows for robust identification and quantification of unique genotypes within a heterogeneous population using standard Sanger sequencing. Our findings demonstrate that the longstanding trade-off between the size and quality of transformed yeast libraries can be overcome. By employing the HEEL method, large DNA libraries can be transformed into yeast with high-efficiency, while maintaining high library quality, essential for successful mutant screenings. This advancement holds significant promise for the fields of molecular biology and protein engineering.

**IMPORTANCE:**

With the recent expansion of artificial intelligence in the field of synthetic biology, there has never been a greater need for high-quality data and reliable measurements of phenotype-to-genotype relationships. However, one major obstacle to creating accurate computer-based models is the current abundance of low-quality phenotypic measurements originating from numerous high-throughput but low-resolution assays. Rather than increasing the quantity of measurements, new studies should aim to generate as accurate measurements as possible. The HEEL methodology presented here aims to address this issue by minimizing the problem of multi-plasmid uptake during high-throughput yeast DNA transformations, which leads to the creation of heterogeneous cellular genotypes. HEEL should enable highly accurate phenotype-to-genotype measurements going forward, which could be used to construct better computer-based models.

## INTRODUCTION

Numerous studies have investigated the molecular mechanisms that affect DNA transformations in both bacteria and yeast. In the case of chemical transformation of bacteria, the primary parameters of importance are temperature (heat-shock), the presence of certain metal ions (rubidium, manganese, and calcium) and/or pH, as reviewed in reference 1. Most studies conclude that chemical transformation induces either a semi-permeabilized and/or transiently permeable membrane structure, or a lowering of the membrane potential, which facilitates a passive DNA uptake (1). Temperature (heat-shock) is also an important factor that stimulates extracellular DNA uptake during chemical transformation of yeast (2). However, unlike for bacteria, yeast chemical DNA transformation is currently understood to be mediated by endocytosis pathways (3), which are strongly enhanced by chemical agents, such as lithium acetate, crowding agents (PEG), and nonspecific single-strand carrier DNA (ssDNA) (2, 3). In the case of bacterial and yeast DNA transformations mediated by electroporation, our current understanding suggests that a passive DNA diffusion through transiently formed pores in the membrane is the primary route of DNA uptake during electroporation (1, 4). Nevertheless, yeast electroporation requires a more complicated and cumbersome protocol, compared with bacterial electroporation, because the rigid yeast cell wall and/or membrane first needs to be conditioned to allow for adequate DNA uptake (5).

Clear cross-species comparisons of DNA transformations are challenging, as different factors affect the DNA uptake in bacteria and yeast. In addition, the convention to present DNA transformation outcomes as transformation efficiencies, by normalizing the number of transformed cells per µg DNA, makes direct comparisons exceptionally difficult and occludes the more important information regarding the maximum library size that can be created in each host. For bacterial DNA transformations, very small amounts of DNA are usually used, often in the range of 10–50 ng (6). Thus, when the number of transformed cells is normalized to µg of DNA, the transformation efficiency gets inflated by a factor of approximately 2-log. While for yeast transformations, which often use 1–10 µg of DNA, the DNA transformation efficiency becomes deflated by as much as one order of magnitude when normalized (5). Consequently, a reported transformation efficiency (CFU/µg DNA) of 10^10^ for *Escherichia coli* means that only 10^7^ cells were transformed with 10 ng DNA per reaction. Yet, a reported transformation efficiency of 10^6^ for yeast would also mean that 10^7^ cells were transformed, but with 10 µg per reaction. Transformation efficiencies are also easily manipulated by changing the amount of transformed DNA, since the number of transformed cells does not corelate linearly with the amount of DNA added during a transformation, and small amounts of DNA can be transformed to reach an exceptionally high transformation efficiency (4). Also, since most studies and synthetic biology endeavors have little-to-no interest in transformation efficiencies *per se*, it is preferable and more accurate to instead report on transformation yields, i.e., the total number of transformed cells per reaction. Another important factor to consider is the fraction of transformed cells in a solution, i.e., how many cells are needed to achieve the desired transformation yield. In this study, we have chosen to exclusively focus on these two parameters to best convey the true value of our results and present our findings in an easily understood and relevant format.

Transformation yields are critical when creating high-diversity mutant libraries for, e.g*.*, directed evolution purposes. However, most chemical transformation methods of either bacteria or yeast are not sufficiently high-throughput to successfully transform high-diversity libraries (1, 2). In contrast, standard electroporation protocols are often 1–2-log more efficient than most chemical transformation methods (1, 4, 5). Thus, for high-throughput random protein engineering, the transformation of high-diversity mutant libraries via electroporation is currently the most effective method. Moreover, while many studies have identified numerous important parameters that affect transformation efficiencies, few studies have identified factors that control multiplicity of DNA transformations (i.e., the number of DNA molecules that enter each cell). The few studies that have investigated this phenomenon have all concluded that the likelihood of transforming a bacterial or yeast cell with multiple DNA molecules is directly correlative with the concentration of extracellular plasmid DNA that is added to the transformation reaction (6–8). This creates a challenging scenario, as transformation yields in both yeast and bacteria also increase with an increased concentration of extracellular plasmid DNA (4). Thus, with current electroporation methodologies, it is not possible to design a strategy that results in both a high fraction of mono-transformed cells (good library quality), while simultaneously also retaining the maximum possible number of transformed cells (transformation yield). This inverse relationship between library size and quality is a tremendous problem for most synthetic biology approaches that rely on high-diversity mutant libraries, as the likelihood of identifying a beneficial mutant within any library is limited by the size, i.e., coverage, of the library. However, what is even more crucial to any library screening effort is the ability to measure and assign a clear phenotype-to-genotype relationship for each individual cell. Thus, library quality and library size are two mutually exclusive factors of equal importance. Moreover, with the increasing use of artificial intelligence (AI) and machine learning algorithms, there is now a great need for precise phenotype-to-genotype measurements to better understand the sequence space of the respective protein. The resulting high-quality data sets can then be used to train computer-based models with the aim of improving accuracy and the insight of subsequent predictions (9).

In this study, we have aimed to improve the transformation efficiency of circular DNA transformations in yeast, while simultaneously maximizing the fraction of mono-transformed cells to achieve the largest possible high-quality mutant library of easy-to-screen yeast cells. To validate our approach, we have designed an automated yeast genotype workflow based on a single-nucleotide polymorphism (SNP) and high-diversity (10N) dual-barcoding approach that allows us to rapidly, and efficiently, identify and quantify the number of plasmids transformed in each single-cell derived yeast colony. Using this dual-barcoding approach, we discovered that subjecting yeast cells to a heat treatment before electroporation increased the fraction of mono-transformed cells from 20% to almost 70% of the transformed yeast population. Remarkably, this heat pretreatment increased the fraction of mono-transformed yeast cells without affecting the transformation yield, which allowed us to retain a transformation yield of >10^7^ yeast cells per electroporation reaction. Thus, we can overcome the classical dilemma of inversely correlated library size and library quality and maintain maximal library size while simultaneously increasing library quality, simply by introducing a heat pretreatment.

## RESULTS

### *Saccharomyces cerevisiae* cells exhibit a low and highly variable survivability post-electroporation when transformed with an established DNA transformation protocol

**Fig 1:**
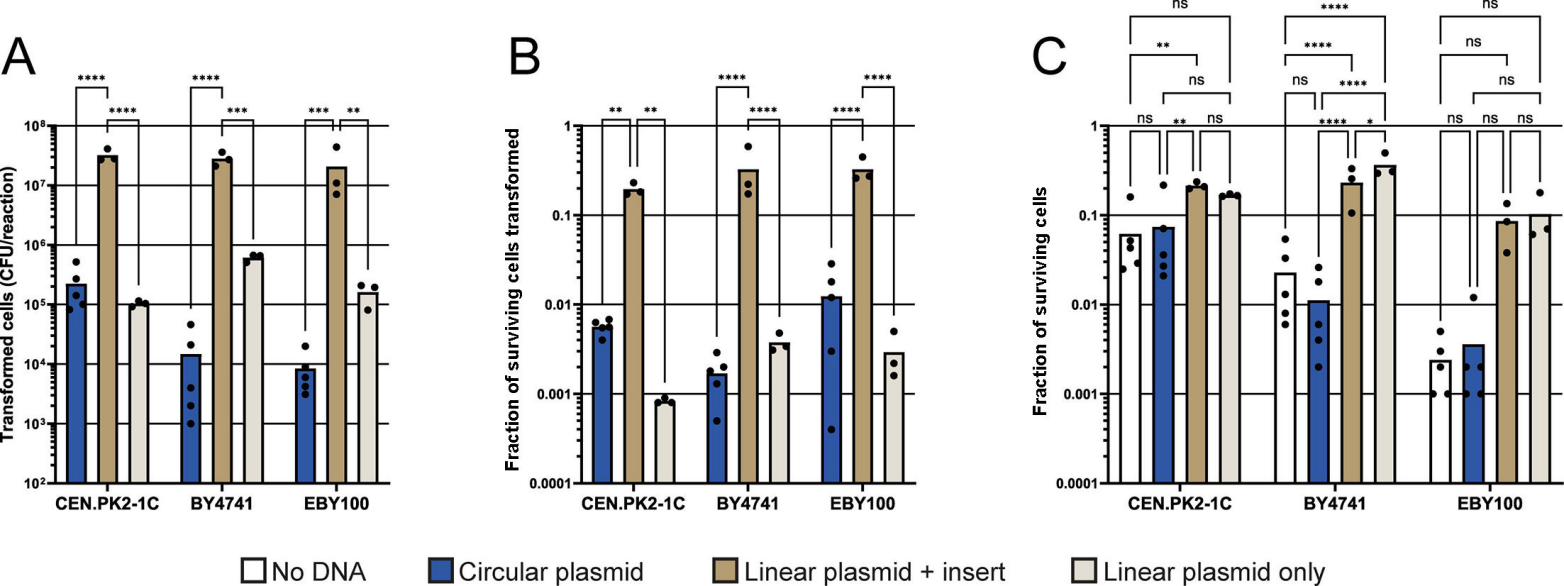
*S. cerevisiae* strains CEN.PK2-1C, BY4741, and EBY100 have low and highly variable survivability post-electroporation when transformed with a previously established protocol (5). Three different *S. cerevisiae* strains were transformed with 8 µg of a circular empty plasmid (pYB-Dual [Leu^+^]), 8 µg of FD XhoI and FD BamHI (Thermo Fisher Scientific) linearized and alkaline phosphatase (Thermo Fisher Scientific) dephosphorylated empty plasmid (pYB-Dual [Leu^+^]) or 8 µg of a linearized and dephosphorylated empty plasmid (pYB-Dual [Leu^+^]) with 24 µg of a PCR-amplified *HIS3* selection marker (1:3 ratio [wt/wt]) designed with 40 bp of homology to either end of the FD XhoI and FD BamHI (Thermo Fisher Scientific) cleaved pYB-Dual plasmid, using the method described by Benatuil et al. (*n* = 3–5) (5, 10). Briefly, yeast cells were conditioned in 100 mM lithium acetate and 10 mM DTT at 30°C for 30 min, then 4–9 × 10^8^ yeast cells per reaction were electroporated at 2.5 kV in a buffer containing 1 M sorbitol and 1 mM CaCl_2_. (**A**) Number of transformed yeast cells per reaction. (**B**) Fraction of surviving yeast cells transformed. (**C**) Total fraction of surviving cells post-electroporation. Statistical significance was calculated by two-way ANOVA with ns: *P* > 0.05, *: *P* ≤ 0.05, **: *P* ≤ 0.005, ***: *P* ≤ 0.0005, and ****: *P* ≤ 0.0001.

Our initial aim was to design a barcoding approach that could be used to easily track both the unique genotype of each DNA molecule within a high-diversity mutant library and to quantify the multiplicity of transformations when transforming our model yeast, *Saccharomyces cerevisiae* (CEN.PK2-1C or CEN.PK110-10C). Toward that end, we first attempted to use a well-known yeast DNA electroporation protocol from Benatuil et al. to achieve ultra-high-diversity libraries in our CEN.PK2-1C yeast strain (5). To our surprise, we were not able to achieve transformation yields as reported by Benatuil et al. When we transformed our CEN.PK2-1C strain with 8 µg of a circular plasmid (pYB-Dual [10]) we observed a transformation yield of approximately 10^5^ transformed cells per reaction (Fig. 1A and B). This is a stark contrast to the previously reported transformation yield of approximately 5 × 10^8^ transformed cells per reaction (5). However, Benatuil et al. performed a gap-repair reaction by transforming yeast with a linear plasmid and a linear insert DNA that had homology with the linear plasmid (5). Linear DNA with homology arms has previously been shown to transform yeast better than both linear DNA without homology arms and circular DNA (11). Thus, we hypothesized that DNA topology issues could be the reason we observed a low transformation yield (Fig. 1A and B). Additionally, Benatuil et al. transformed the common *S. cerevisiae* yeast-surface display strain EBY100 in their study, not CEN.PK2-1C. Thus, we thought that intra-strain differences might also contribute to the low transformation yield we observed (Fig. 1A and B) (5). To explore this possibility, we repeated our transformation experiment with both EBY100 and the common *S. cerevisiae* strain BY4741, using both circular and linear DNA (Fig. 1A and B). Circular DNA transformations of both BY4741 and EBY100 were even less efficient than CEN.PK2-1C, with approximately 10^4^ transformed cells per reaction (Fig. 1A and B). In contrast, all three investigated yeast strains exhibited transformation yields that were either 2-log (CEN.PK2-1C) or 3-log higher (BY4741 and EBY100) when 8 µg of a linear plasmid was transformed, along with 24 µg of a linear DNA insert with 40 bp homology to each end of the linear plasmid (Fig. 1A and B). Yet, while we could transform approximately 1–3 × 10^7^ yeast cells per reaction using linear plasmid DNA, this is still >1 log lower than the approximately 5 × 10^8^ transformed cells per reaction reported by Benatuil et al. (5). However, this discrepancy has also been reported in multiple other studies (12–14). These studies have all reported transformation yields similar to what we observe (10^6^–10^7^ transformants per reaction) when they tried to reproduce the protocol by Benatuil et al. and transform yeast cells with linear plasmid DNA (Fig. 1A and B) (5, 12–14). Unfortunately, due to the lack of molecular details in the original study, it was impossible for us to attempt to reproduce the findings by Benatuil et al. any further (5). Additionally, we observed a highly inconsistent yeast cell viability post-electroporation when using the protocol by Benatuil et al. (Fig. 1C) (5). We observed a trend that indicated that a greater number of yeast cells survived the electroporation reaction when transformed with linear DNA, rather than with circular DNA, or no DNA. Although, because of the variability that we observed between individual transformation reactions, most of these differences were not statistically significant (Fig. 1C). We believe that the possible increased survivability of yeast cells we observe when linear plasmid DNA is transformed might not be due to the DNA itself, but rather the effect of the volume of the DNA solution. When we added our linear plasmid DNA solution to our yeast electroporation reaction, it constituted almost 10% of the total volume of the electroporation reaction. This was because of the difficulty in preparing a linearized and dephosphorylated plasmid DNA solution of high concentration. Thus, we believe that by diluting the electroporation reaction, we changed the concentration of the buffer, i.e*.*, sorbitol and CaCl_2_, and added a high concentration of trace salts from the DNA purification kit. This could have changed the conditions of the electroporation reaction, thus allowing for a greater yeast survivability. We concluded that the electroporation protocol described by Benatuil et al. was highly specialized for linear DNA transformations and was also prone to result in a highly variable yeast survivability (5).

### Efficient *S. cerevisiae* circular DNA transformation by electroporation requires single-stranded carrier DNA and lower voltage

**Fig 2:**
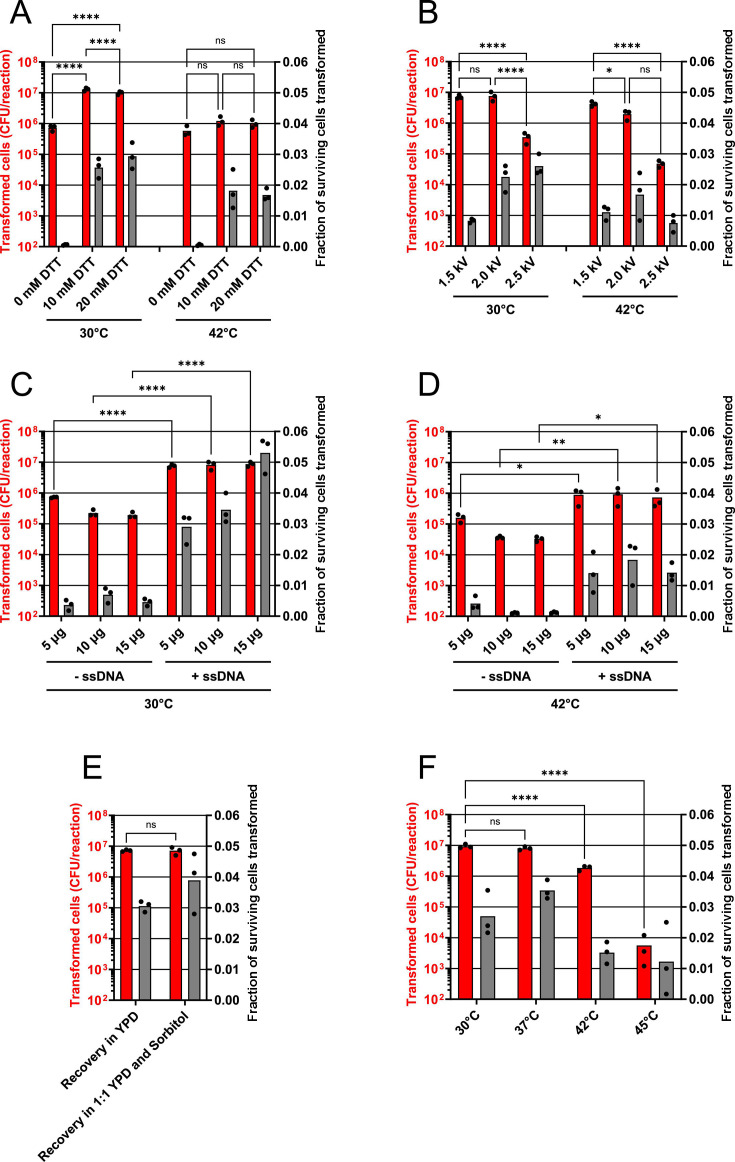
The DNA transformation method by Benatuil et al. can be optimized to allow for efficient circular DNA transformations by changing the electroporation voltage and by adding single-strand salmon sperm DNA (ssDNA) to each transformation reaction. *S. cerevisiae* strain CEN.PK110-10C was transformed with 5 µg of a pRS413 (His^+^) circular plasmid molecule and 100 µg of salmon sperm single-strand DNA (ssDNA), using a preliminary HEEL DNA transformation workflow. Briefly, yeast cells were conditioned in 100 mM lithium acetate and 20 mM DTT at 30°C or 42°C for 60 min, then electroporated at 2.0 kV in a buffer containing 1 M sorbitol and 1 mM CaCl_2_, unless otherwise stated. (**A**) The effect on yeast transformability by changing the DTT concentration, (**B**) the electroporation voltage, (**C and D**) plasmid DNA concentration and adding ssDNA, (**E**) omitting sorbitol from the recovery media, or (F) changing the heat-shock temperature (*n* = 3). Statistical significance was calculated by two-way ANOVA with ns: *P* > 0.05, *: *P* ≤ 0.05, **: *P* ≤ 0.005, ***: *P* ≤ 0.0005 and ****: *P* ≤ 0.0001.

**Fig 3:**
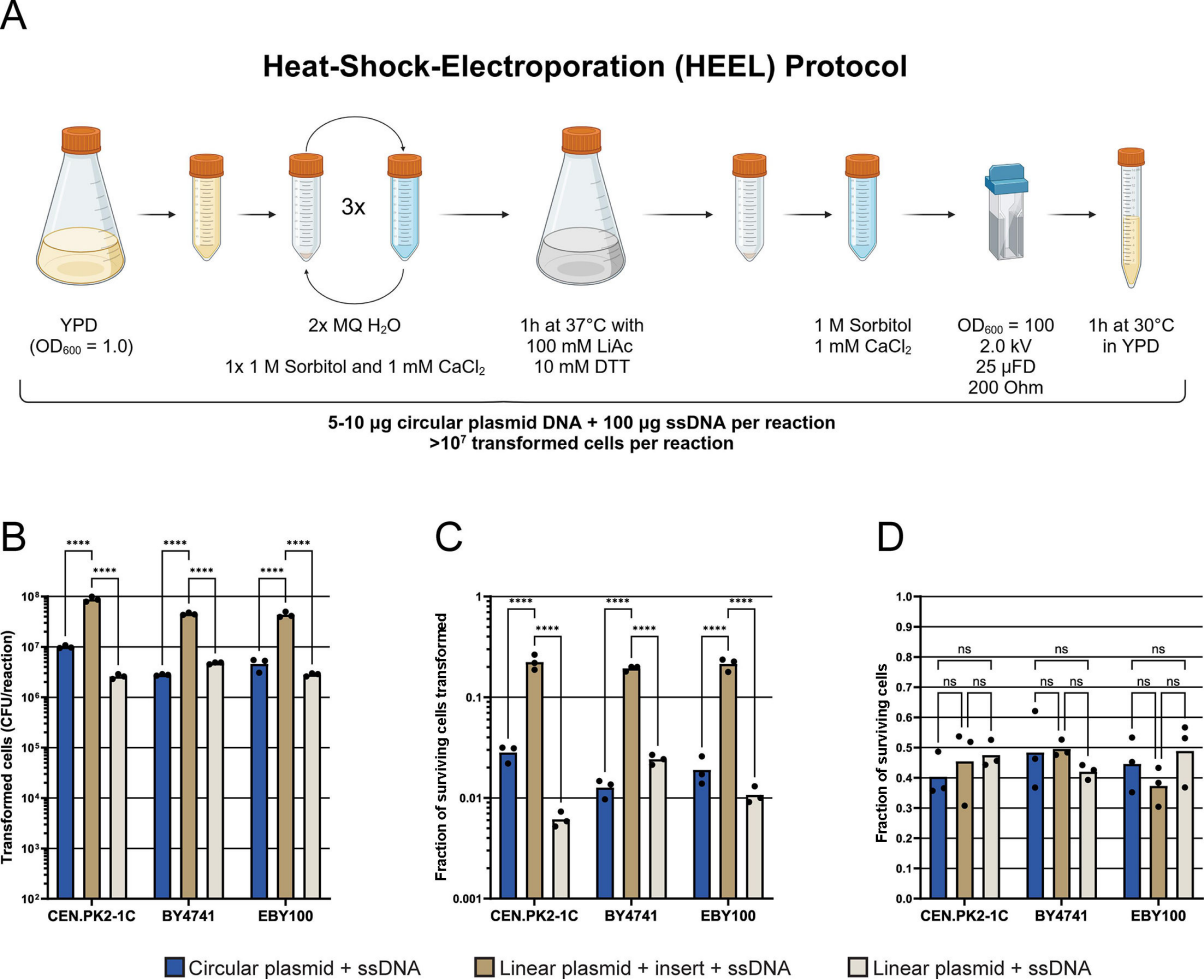
Overview of the HEEL methodology. (**A**) Schematic illustration of the heat-shock-electroporation (HEEL) yeast circular DNA transformation method. Illustration created with BioRender.com. (**B–D**) Three different *S. cerevisiae* strains were transformed with 100 µg of ssDNA and either 5 µg of a circular empty plasmid (pYB-Dual [Leu^+^]), 5 µg of a FD XhoI and FD BamHI (Thermo Fisher Scientific) linearized and alkaline phosphatase (Thermo Fisher Scientific) dephosphorylated empty plasmid (pYB-Dual [Leu^+^]), or 5 µg of a linearized and dephosphorylated empty plasmid (pYB-Dual [Leu^+^]) with 5 µg of a PCR-amplified *HIS3* selection marker (1:5 mole ratio) designed with 40 bp of homology to either end of the FD XhoI and FD BamHI (Thermo Fisher Scientific) cleaved pYB-Dual plasmid, using the HEEL DNA transformation method (*n* = 3) (10). Briefly, yeast cells were conditioned in 100 mM lithium acetate and 10 mM DTT at 37°C for 60 min, then 4–9 × 10^8^ yeast cells per reaction were electroporated at 2.0 kV in a buffer containing 1 M sorbitol and 1 mM CaCl_2_. (**B**) Number of transformed yeast cells per reaction. (**C**) Fraction of surviving yeast cells transformed. (**D**) Total fraction of surviving cells post-electroporation. Statistical significance was calculated by two-way ANOVA with ns: *P* > 0.05, *: *P* ≤ 0.05, **: *P* ≤ 0.005, ***: *P* ≤ 0.0005 and ****: *P* ≤ 0.0001.

In our efforts to reproduce the DNA transformation method described by Benatuil et al., we realized that preparing and purifying enough linearized and dephosphorylated plasmid DNA, as well as sufficient PCR-amplified insert-DNA, was not a trivial matter (5). We also observed that more than 10^5^ yeast cells could be transformed by a linearized and dephosphorylated plasmid DNA molecule (Fig. 1A and B). Thus, linear DNA transformations are not only more labor-intensive, but they are also more prone to creating lower quality yeast libraries with a high amount of empty vector background. To address this, we proceeded to investigate the parameters that would allow for a highly efficient transformation of circular plasmid DNA. As we observed very low and inconsistent yeast survivability post-electroporation when using the DNA transformation method described by Benatuil et al*.,* we concluded that the conditions described in this transformation protocol are too harsh on the cells (5). Following this line of thought, we chose to continue with our preferred model strain CEN.PK110-10C. This strain is closely related to the CEN.PK2-1C strain, which displayed the greatest transformation yield (10^5^) when transformed with a circular plasmid molecule (Fig. 1A and B). Next, we established a permissive electroporation protocol, which we confirmed was the optimal approach, by systematically changing one parameter at a time (Fig. 2A through F). Using this optimized protocol (see Material and Methods), we could transform approximately 3% of all surviving cells in a yeast cell solution and achieve a transformation yield of >10^7^ cells per reaction (Fig. 3A and B). From our investigations, we could confirm that both the DTT (10–20 mM) and DNA concentration (4–8 µg) previously reported by Benatuil et al. were also optimal when transforming circular DNA (Fig. 2A, C and D) (5). However, we could not confirm the importance of any additional parameters previously reported (5). When we changed the electroporation voltage from 2.5 to 2.0 kV, almost 1-log more cells were transformed, possibly because the survivability of the yeast increased from approximately 1% to 26% of all yeast cells (Fig. S1B). While an electroporation voltage of 1.5 kV did allow for even more cells to survive the treatment (63%), it did not increase the transformation yield compared with 2.0 kV (Fig. S1B). Thus, we observed that a greater fraction of viable cells was transformed at 2.0 kV compared with 1.5 kV, which is a parameter of great importance for selection-free library screenings. We also observed that recovering cells after transformation in a 50% sorbitol and 50% YPD solution did not significantly influence survivability or the transformation yield (Fig. 2E; Fig. S1E) (5). Additionally, we theorized that if chemical transformation of yeast is stimulated by ssDNA, then this might also hold true for yeast when electroporated (2). As expected, the addition of 100 µg of ssDNA to the electroporation reaction increased the transformation yield >1 log when we transformed our CEN.PK110-10C strain with a circular pRS413 plasmid (Fig. 2C and D) (15). Recently, Loock et al. have reported that ssDNA also increases linear DNA transformation yields during *S. cerevisiae* electroporation (16). Thus, we observed that many parameters important for chemical transformation of yeast (Lithium acetate and ssDNA) are also important for electroporation of yeast. Consequently, we were curious to investigate if heat-shocking yeast cells would also affect the transformation yield during electroporation. However, when we heat-shocked yeast cells at 42°C before electroporation, we only observed reduced transformation yields (Fig. 2A through C and F). Changing the electroporation voltage to 1.5 kV slightly compensated for this diminished transformation yield, but not to a point where we could justify a potential advantage of exposing yeast cells to a 42°C heat-shock step before electroporation (Fig. 2B). Interestingly, heat-shocking cells at 37°C before electroporation did not diminish transformation yields, although we did observe a potential increase in the fraction of transformed cells as only 23% of cells survived the mild heat-shock treatment, compared with 31% when the heat-shock was omitted (Fig. 2F; Fig. S1F). Thus, we concluded that a mild heat-shock before electroporation was an easy and convenient way of increasing the fraction of surviving cells being transformed, and we named this new method heat-shock-electroporation (HEEL).

### Heat-shock-electroporation (HEEL) allows for circular DNA to be transformed into *S. cerevisiae* with comparable efficiency to linear DNA

Using our new yeast transformation methodology, we were curious to investigate if HEEL could increase the efficiency of circular or linear plasmid DNA transformations for all three of the previously investigated yeast strains (Fig. 1A through C). When we used the HEEL methodology to transform CEN.PK2-1C, BY4741 or EBY100 with 100 µg of ssDNA and 5 µg of a circular plasmid, we observed an approximately 2-log increase in the transformation yield as compared to what we previously had observed when using the protocol by Benatuil et al., regardless of which strain was transformed (Fig. 1 and 3) (5). Of the three yeast strains investigated, CEN.PK2-1C continued to exhibit the highest affinity for circular DNA transformations (Fig. 3B and C). In contrast, when we transformed the three yeast strains with 100 µg of ssDNA, 5 µg of a linear plasmid and 5 µg of linear insert DNA (1:5 mole ratio) with 40 bp homology to each end of the linear plasmid, we observed less than a threefold increase in the transformation yield compared to what we previously had observed when using the protocol by Benatuil et al., regardless of which strain was transformed (Fig. 1A and B, 3B and C). However, we did observe a strong increase in the amount of empty vector background as more than 10^6^ yeast cells could be transformed by the linearized and dephosphorylated pYB-Dual plasmid (Fig. 3B and C). We suspect that this increased empty vector background might be the result of non-specific ssDNA acting as a potential bridge for linear DNA re-circularization *in vivo*. Collectively, these findings demonstrate that the HEEL methodology enables the efficient transformation of yeast cells with high-quality circular DNA libraries that can be created with little-to-no empty vector background. Thus, HEEL allows for circular DNA transformation yields that are comparable to high-throughput DNA transformation methods optimized for linear DNA and allows for the direct transformation of yeast with non-modified DNA libraries extracted from bacterial cells.

### A dual-barcode approach allows for direct enumeration of plasmids post-transformation using Sanger sequencing

**Fig 4:**
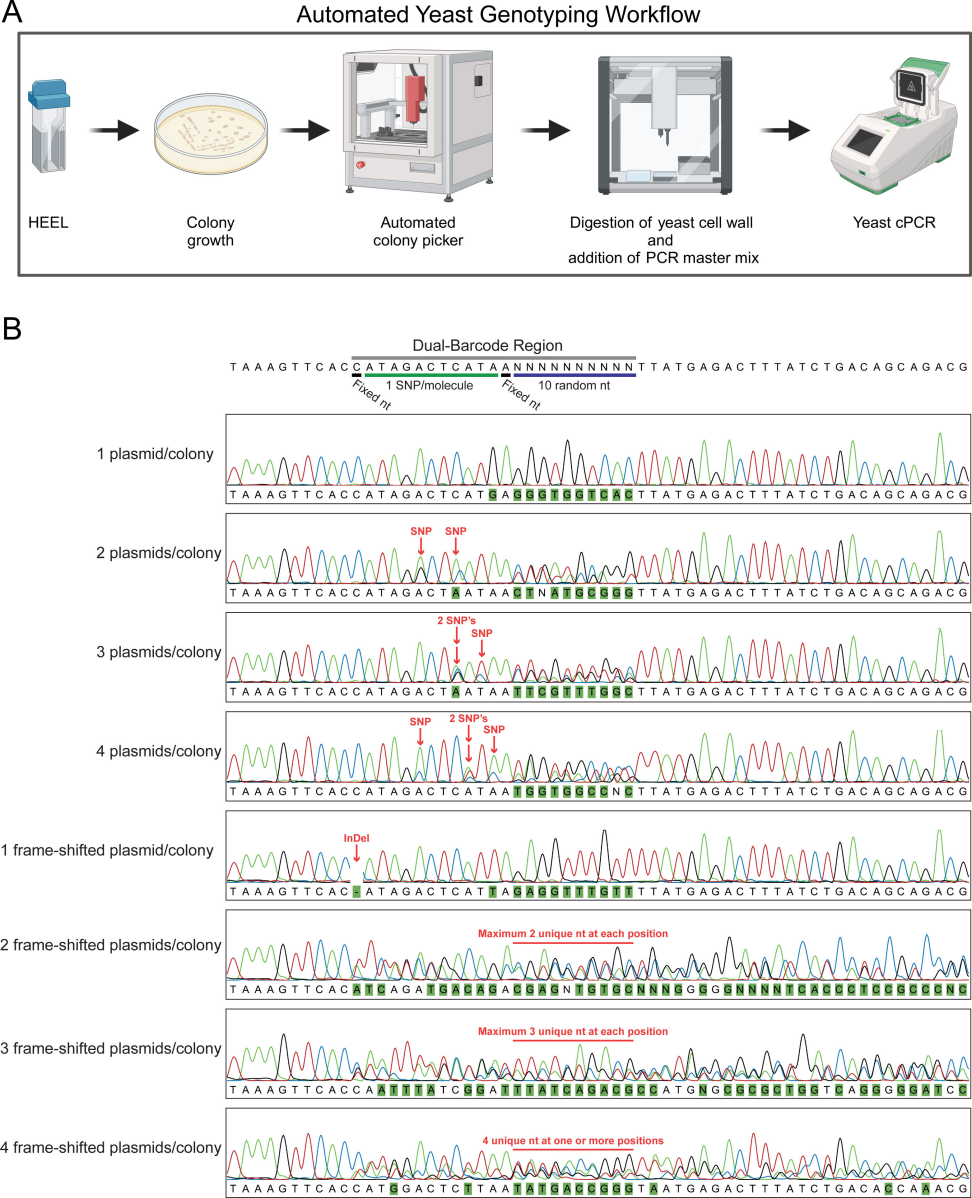
A dual-barcode approach can enumerate the number of plasmid molecules present in each single-cell-derived yeast transformant, using a single Sanger sequencing reaction. (**A**) Schematic illustration of our automated yeast genotyping workflow. Illustration created with BioRender.com. (**B**) Schematic illustration of the dual-barcode region and representative Sanger sequencing chromatograms from single-cell-derived yeast colony PCR (cPCR). Demonstration of the utility of both the SNP and high-diversity region of the dual-barcode to enumerate the number of unique sequences in a mixed population. Green boxes indicate variable nucleotides, which do not match the reference sequence.

Multiplicity of transformation in yeast during high-throughput DNA transformations have previously been investigated (7). During this investigation, Scanlon et al. reported that when transformation yields were high (9.3 × 10^6^ transformed cells per reaction), 88% of all yeast cells were transformed with multiple unique DNA molecules (7). Likewise, Benatuil et al. reported that 85% of all yeast cells were transformed with multiple unique plasmid molecules when they used their high-throughput yeast DNA transformation method (5). As HEEL was developed to improve the efficiency of circular DNA transformations, we were curious to investigate how our method affected yeast multiplicity of transformation compared to the above-mentioned studies, which both used linear DNA. In their study, Scanlon et al. used a plasmid library consisting of a 3nt random barcode to identify how many unique plasmids were transformed into each yeast cell during electroporation (7). However, to quantify the number of unique sequences per cell, Scanlon et al. first performed an outgrowth of the transformed yeast cells for up to 35 h and then identified the number of unique plasmid sequences they could find in 10–20 randomly selected and sequenced colonies. Statistical probabilities were then applied to the findings to determine the most probable number of plasmids per cell (7). This approach allowed Scanlon et al. to determine that most cells (88% of all transformed cells) were transformed with a median of four plasmids per cell (7). Drawing inspiration from Scanlon et al*.,* we designed a more comprehensive method of quantifying the multiplicity of transformation in yeast using a dual-barcode approach. This dual barcode was designed to contain one region of 11 bp, which only contained one SNP per molecule and one completely random 10 nt region (Fig. S2A). Using this dual barcode, we could directly determine the number of plasmids that each yeast cell was transformed with during an electroporation by simply performing a single Sanger sequencing reaction on the region encoding the dual barcode and then enumerating the number of unique SNPs found during sequencing. Although it should be noted that accurate SNP calling for a Sanger sequencing reaction requires that at least 15% of all sequences are identical, i.e*.*, the maximum number of unique SNPs (plasmids) that can be identified during a single Sanger sequencing using an SNP-based method is 5–6 per cell (17). Furthermore, as no library creation method is perfect, we expected to have both empty vector contamination as well as unwanted nucleotide deletions within a few of the barcoded plasmids we created for our library. To overcome this obstacle, we designed the second 10 nt random region within the dual barcode to act as a quality control and complementary method to the SNP base-calling approach (Fig. S2A). This 10 nt random region was designed to exploit the statistically highly unlikely event that all 10 random nucleotides were identical if multiple unique plasmids were randomly selected (Fig. S2B). If a cell was transformed with two or three plasmids containing a random 10 nt region, then this region should contain a maximum of two or three unique nucleotides, respectively, at every position. While if a cell contains four plasmids, then at least one position should contain all four bases. The statistical likelihood of accurately quantifying the presence of one, two, or three plasmids per cell using this approach is >99%, while cells transformed with four plasmids will be enumerated correctly approximately 63% of times (Fig. S2B). Though this method cannot be used to determine the exact genotype of each plasmid within a library, and only has a maximum detection limit of four unique plasmids per single-cell-derived yeast colony, it functions well as an accurate, but low-resolution, complementary methodology to the SNP-based barcoding approach (Fig. S2B). In the case of the 11 nt barcode, where only one SNP per molecule is present, only 33 unique sequences (11 nt × three possible alternative nucleotides per position) can be created (Fig. S2A). The likelihood that two, three, four, five, or six plasmids would have the exact same SNP is 3%, 9%, 17%, 27%, and 38% respectively (Fig. S2B). Thus, while the high-diversity barcode (10N) allows for a more accurate quantification of multiplicity of plasmids in the 1–3 plasmids per cell range, the SNP-based barcode allows for a more accurate quantification in the 4–6 plasmids per cell range, as well as a precise SNP (genotype) determination (Fig. S2B). To further streamline the plasmid quantification process, we also designed an automated yeast genotyping pipeline, which allowed us to leverage the efficiency of an automated colony picker and a liquid-handling robot to quickly collect, prepare, PCR amplify, and then sequence the mini-libraries found within each single-cell-derived yeast colony (Fig. 4A) (see Material and Methods).

**Fig 5:**
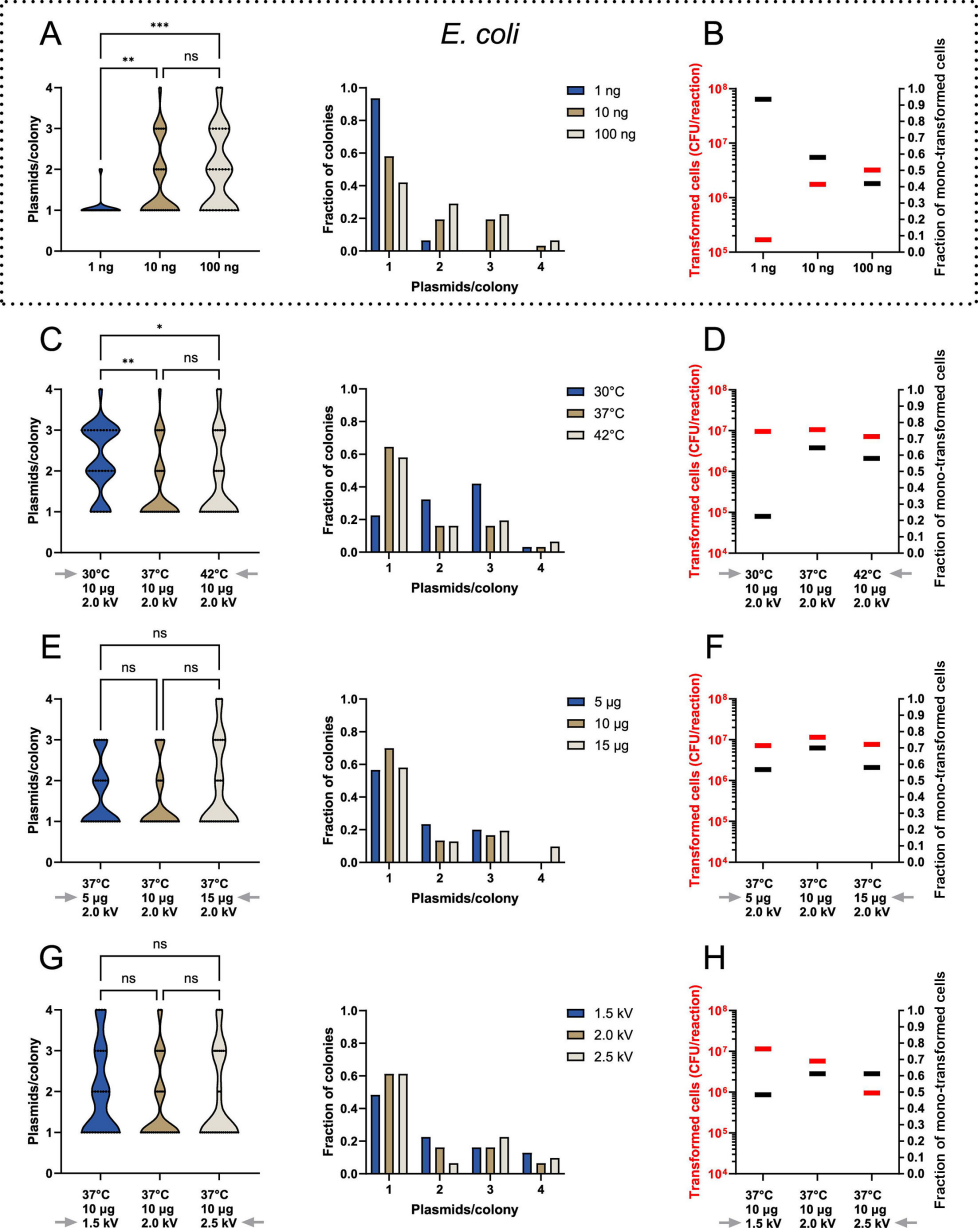
A mild heat treatment before electroporation increases the fraction of mono-transformed yeast cells, without diminishing transformation yields. (**A**) Plasmid enumeration of single-cell derived *E. coli* TOP10 bacterial colonies, transformed with different concentrations of a pUC19 dual-barcoded plasmid library (*n* = 31). (**B**) Correlation between transformation yields and the fraction of mono-transformed *E. coli* cells during an electroporation, as a result of the plasmid DNA concentration used. The fraction of mono-transformed cells was calculated from data in panel A. (**C–H**) Plasmid enumeration of single-cell derived *S. cerevisiae* strain CEN.PK110-10C transformed with a pRS413 (His^+^) dual-barcoded plasmid library using the HEEL method. Changing only the (C) heat-shock temperature (*n* = 31), (**E**) plasmid DNA concentration (*n* = 30–31), or (G) electroporation voltage (*n* = 31). Correlation between transformation yields and the fraction of mono-transformed yeast cells as a result of the (D) heat-shock temperature, (**F**) plasmid DNA concentration, or (H) electroporation voltage used. The fraction of mono-transformed yeast cells was calculated from data in panels C, E, and G, respectively. Statistical significance was calculated by two-way ANOVA with ns: *P* > 0.05, *: *P* ≤ 0.05, **: *P* ≤ 0.005, and ***: *P* ≤ 0.0005.

Multiplicity of plasmid transformations in *E. coli* has previously been shown to correlate with the DNA concentration used during a transformation (6, 8). Thus, to validate our dual-barcode approach, we created a small pUC19 plasmid library, containing the dual-barcoded region, which we then transformed into the *E. coli* cloning strain TOP10 (Fig. 5A and B). We added 1, 10, or 100 ng of our plasmid DNA library to each electroporation reaction. Post-electroporation, we colony PCR amplified the barcoded region from all plasmids found within 31 individual single-cell-derived bacterial colonies per experiment. This high number of biological replicates was chosen to ensure robust statistical reliability in all statistical tests. PCR-amplified DNA, containing the barcoded region of each pUC19 plasmids, was Sanger sequenced and manually curated for the presence of multiple SNPs. From this analysis, we observed that when our TOP10 cells were electroporated with 1 ng of plasmid DNA, then 93% of all colonies contained one unique plasmid sequence (Fig. 5A). However, only 58% and 42% of cells were mono-transformed when 10 ng or 100 ng of DNA were used, respectively (Fig. 5A). However, while 1 ng yielded the highest quality library, it also resulted in >1 log fewer transformed bacterial cells per reaction compared to when 10 ng or 100 ng of plasmid DNA was electroporated (Fig. 5B). These results support previous findings, which have demonstrated that increasing extracellular DNA concentrations during electroporation of *E. coli* increases transformation yields, but at the expense of decreasing the fraction of mono-transformed cells (8).

### A mild heat treatment before electroporation increases the fraction of mono-transformed yeast cells without diminishing transformation yields

Armed with the knowledge that our dual-barcode approach allowed for a fast and efficient quantification of the multiplicity of DNA transformations, we recombined our pUC19 plasmid library with a pUC19-based yeast shuttle vector (pRS413) *in vivo* and then proceeded to transform our CEN.PK110-10C model strain with our new pRS413 yeast plasmid library. We already observed that treating yeast cells with a mild 37°C heat-shock prior to electroporation resulted in no reduction in the transformation yield, even though fewer cells survived the treatment (Fig. 2F; Fig. S1F). Thus, we were curious to investigate if this increased fraction of surviving transformed cells would also translate to an effect on the multiplicity of transformation (Fig. 2F). We subjected our CEN.PK110-10C yeast cells to either a 42°C or 37°C heat treatment, or a 30°C incubation, before electroporation. We then isolated and colony PCR amplified the plasmid-encoded barcodes from 31 individual single-cell-derived yeast colonies per condition to ensure robust statistical reliability in our calculations (Fig. 4A). All 93 yeast colony PCR reactions succeeded in generating an easy to sequence PCR product. From the Sanger sequencing chromatograms, we were able to conclude that approximately 15%–25% of our plasmid library consisted of vectors that lacked >3 nt of the barcoded region, and approximately 10%–15% of the library contained small 1–3 nt deletions within the barcoded sequence. Using a combination of both the SNP and the high-diversity region within the dual-barcode, we were still able to quantify the multiplicity of plasmid transformations for all 93 colonies (Fig. 4B, 5C and D). Similar to previous findings by Scanlon et al., we could also observe that when we used our improved yeast electroporation protocol, and when transformation yields were high (>10^7^ transformed cells per reaction), only 20% of all cells were mono-transformed (Fig. 5C and D) (7). However, we also observed that a mild 37°C heat-shock prior to electroporation drastically increased the fraction of mono-transformed cells, with 61%–70% of all transformed yeast cells being mono-transformed (Fig. 5C through H). Heat-shocking cells at a higher temperature (42°C) did not have an additional beneficial effect. Next, we were curious to investigate if changing the DNA concentration or electroporation voltage could increase this effect even further. Remarkably, neither the fraction of mono-transformed cells nor the transformation yield changed significantly when we heat-shocked cells at 37°C, but either decreased or increased the amount of plasmid DNA to 5 µg or 15 µg, respectively (Fig. 5E and F). Likewise, changing the electroporation voltage to either 1.5 or 2.5 kV did not significantly change the fraction of mono-transformed cells (Fig. 5G and H), although the transformation yield did decrease by >1 log when a voltage of 2.5 kV was used compared with either 1.5 kV or 2.0 kV (Fig. 5G and H). Thus, we concluded that our mild heat-shock and electroporation method could not easily be improved further by changing the physical and chemical parameters investigated in this study. Additionally, since the enrichment of mono-transformed cells through heat-shocking did not reduce the transformation yield, we also concluded that heat-shocking yeast cells before electroporation is a fast, efficient, and exceptionally convenient way of increasing the quality of a transformed yeast DNA library, without sacrificing diversity (Fig. 5C and D).

### MiSeq sequencing validates the dual-barcode- and Sanger-based plasmid enumeration approach

**Fig 6:**
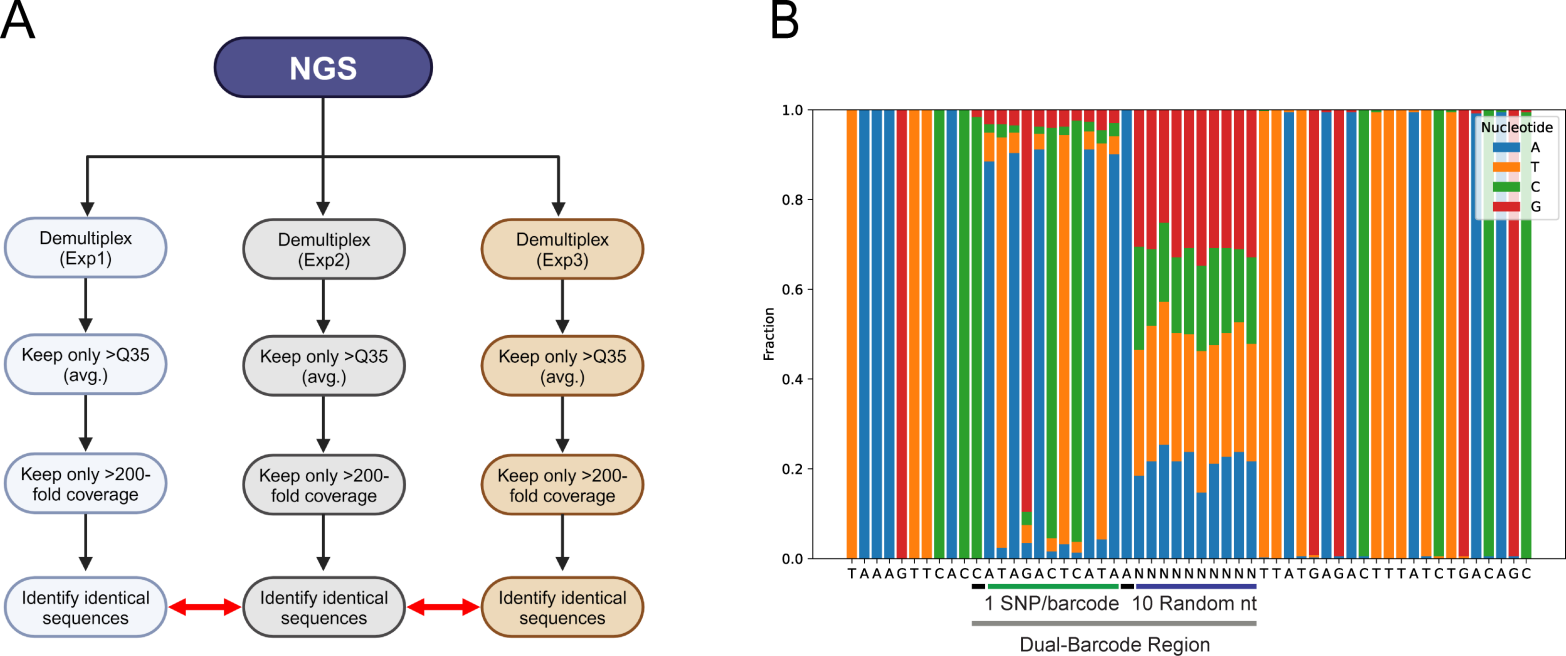
Overview of the NGS workflow. (**A**) Schematic illustration of the NGS workflow used to enumerate the most probable number of unique dual-barcoded sequences in each library (experimental condition). Illustration created with BioRender.com. (**B**) Sequence bias and nucleotide frequency analysis of the dual-barcode region. The analysis includes all NGS-identified dual-barcoded sequences that contain the entire barcoded region.

To validate our approach of using a dual-barcode and simple Sanger sequencing to quantify the multiplicity of plasmid transformation(s) in yeast, we designed our automated yeast colony PCR workflow to also apply Illumina MiSeq P5 and P7 flow-cell binding adapter sequences, along with unique, experiment/condition specific, Illumina i7 barcodes (Fig. 4A). This allowed the same DNA fragments sequenced by Sanger sequencing to also be sequenced by Illumina MiSeq NGS. After the number of unique plasmids was enumerated by conventional Sanger sequencing, the DNA from all 279 individual yeast colony PCRs (31 colonies × 9 conditions), excluding two failed PCRs, was pooled, purified, and sequenced using the Illumina MiSeq platform. From the Sanger sequencing data, we knew that we had a low-diversity library of <1,000 total unique sequences in our pooled MiSeq library. Thus, we hypothesized that with our low-diversity library and the high sequencing coverage we would achieve with an NGS approach, we would regrettably detect even rare mutagenic events and/or sequencing error(s) at a high level, which would lead us to erroneously identify unique barcoded sequences. Some of the possible sources of errors during our NGS analysis would include, but not be limited to, cross-well contamination(s) during the colony PCR sample preparation (a common problem for unspecific metagenomic sequencing [18]), mutations introduced by the Taq DNA polymerase during the PCR amplification of the barcoded region (8 × 10^−6^ mutations/bp/DNA duplication [19]), MiSeq sequencing errors (0.2%–1.2% chance of incorrect base calling, dependent on the sequence context [20, 21]), as well as index hopping during the NGS demultiplexing (0.2%–6% [22]). In the case of index hopping and cross-well contaminations during sample preparations, this would lead to unique barcodes being assigned to the wrong library (experimental conditions), and thus falsely inflate the actual plasmid count for each experimental condition. PCR mutations and MiSeq-dependent sequencing errors would both lead to the creation of additional unique barcoded sequences for each experimental condition, which would also falsely inflate the actual plasmid count per condition. To overcome these issues, we processed, filtered, and curated our NGS data in three steps (Fig. 6A). (i) First, we removed all sequencing reads that had an average base-calling quality of <Q35, removing 38%–43% of all reads per experimental condition. (ii) To best exclude erroneous sequences and remove them from our downstream NGS analysis, we applied a rational cutoff and excluded all sequences that had a coverage of less than 200-fold. This coverage was selected as we observed that a large proportion of unique barcodes had a very low abundance of only 10- to 100-fold coverage (Fig. S3). We concluded that these low-abundant barcodes most likely constituted our PCR, and/or NGS-specific background. (iii) Finally, we performed a sequence similarity analysis where we curated and defined identical or near-identical sequences (only 1 nt difference) found in multiple libraries as “uncertain.” i.e., if identical sequences occurred in two or more libraries (experimental conditions), then one of these sequences would most probably be the result of an index hopping error or from a cross-well contamination event during the sample preparation. Likewise, if two sequences were near-identical, i.e*.*, only differed by 1 nt, then we reasoned that one of these sequences most likely was the result of a PCR or MiSeq sequencing error. The rationale behind this hypothesis was as follows: each dual-barcode sequence can create a maximum of 3.46 × 10^7^ unique sequences (1,048,576 [10N] × 33 possible SNPs) or a minimum of 8.65 × 10^6^ unique sequences (262,144 [9N] × 33 possible SNPs), if 1 nt is allowed to be identical (Fig. S2A). Since we only expected <1,000 random sequences in our NGS data, the likelihood that two unique sequences, or near-identical sequences, simultaneously occurring in multiple libraries/experiments is highly unlikely. However, as we could not determine if these ambiguous sequences should be excluded, because they arose from index hopping or cross-well contamination, or if two similar sequences should be counted as one, if they arose from PCR or sequencing errors, we instead choose to use these uncertain sequences to calculate the range of the most probable number of plasmids per library (Table 1). After we curated our NGS data, we could clearly verify our previous findings, as the MiSeq NGS- and Sanger-based measurements closely correlated with each other (Table 1). For eight of the nine experimental conditions, the Sanger-based plasmid enumeration was within the range of the most probable number of plasmids calculated using the NGS data (Table 1). Although for library 3 (42°C heat-shocked cells), the minimum number of NGS-enumerated unique plasmid sequences was approximately 5% higher than the number of unique plasmids enumerated by Sanger sequencing. Finally, we analyzed if our random dual-barcode library contained any sequence bias that might affect our NGS analysis (Fig. 6B). From this analysis, we could conclude that all four nucleotides could be found at approximately equal abundance at each of the 10 random positions, and in the 11 nt SNP-based barcode we could observe that all three alternative nucleotides existed at every position of the barcode, although at a low abundance (Fig. 6B). As we expected 1 SNP per barcode, and there were 33 possible SNP combinations, this low abundance clearly demonstrated that our barcoding workflow had produced the intended diversity, nucleotide distribution, and sequence composition. Collectively, from these findings, we concluded that our NGS data closely correlated with our Sanger sequencing results and that our dual-barcoding approach allowed for an accurate enumeration of the number of plasmids per yeast cell in the ≤4 plasmids per yeast cell range (Table 1). We could also conclude that regardless of the plasmid enumeration deviation for library 3, our Sanger sequencing and NGS data independently led to the same experimental conclusion, i.e., that a mild heat-shock (37°C) reduced the multiplicity of plasmid transformations in yeast without diminishing the transformation yield.

**TABLE 1:** MiSeq sequencing validates the dual-barcode and Sanger-based plasmid enumeration approach^*b*^

Library	Condition	Sanger sequencing: total number of plasmids	NGS				
			Total number of plasmids	Number of identical sequences	Number of near-identical sequences	Uncertain sequences	Most probable number of plasmids
Exp1	30°C (no heat-shock)	70	94	22	13	35	59–94
Exp2	37°C (mild heat-shock)	49	58	15	7	22	36–58
Exp3	42°C (heat-shock)	54	79	16	6	22	57–79
Exp4	5 µg plasmid DNA	49^*a*^	56^*a*^	18	9	27	29–56
Exp5	10 µg plasmid DNA	44^*a*^	49^*a*^	12	8	20	29–49
Exp6	15 µg plasmid DNA	56	62	15	5	20	42–62
Exp7	1.5 kV electroporation	60	70	17	7	24	46–70
Exp8	2.0 kV electroporation	52	60	14	6	20	40–60
Exp9	2.5 kV electroporation	56	76	15	5	20	56–76
Total number of plasmids		490	604	144	64	208	394–604

^*a*^ Result was calculated from 30 individual yeast colonies, rather than 31.

^*b*^ Comparison of the Sanger- and NGS-derived plasmid enumerations, including the occurrence of identical and near-identical (1 nt difference) sequences within and between different libraries (experimental conditions). Uncertain sequences were subtracted from the NGS-enumerated sequences to obtain a range of the most probable number of plasmids per library.

### HEEL creates DNA libraries with near-perfect phenotype-to-genotype association that is almost 2-log larger than current high-throughput yeast genome integration methods

**Fig 7:**
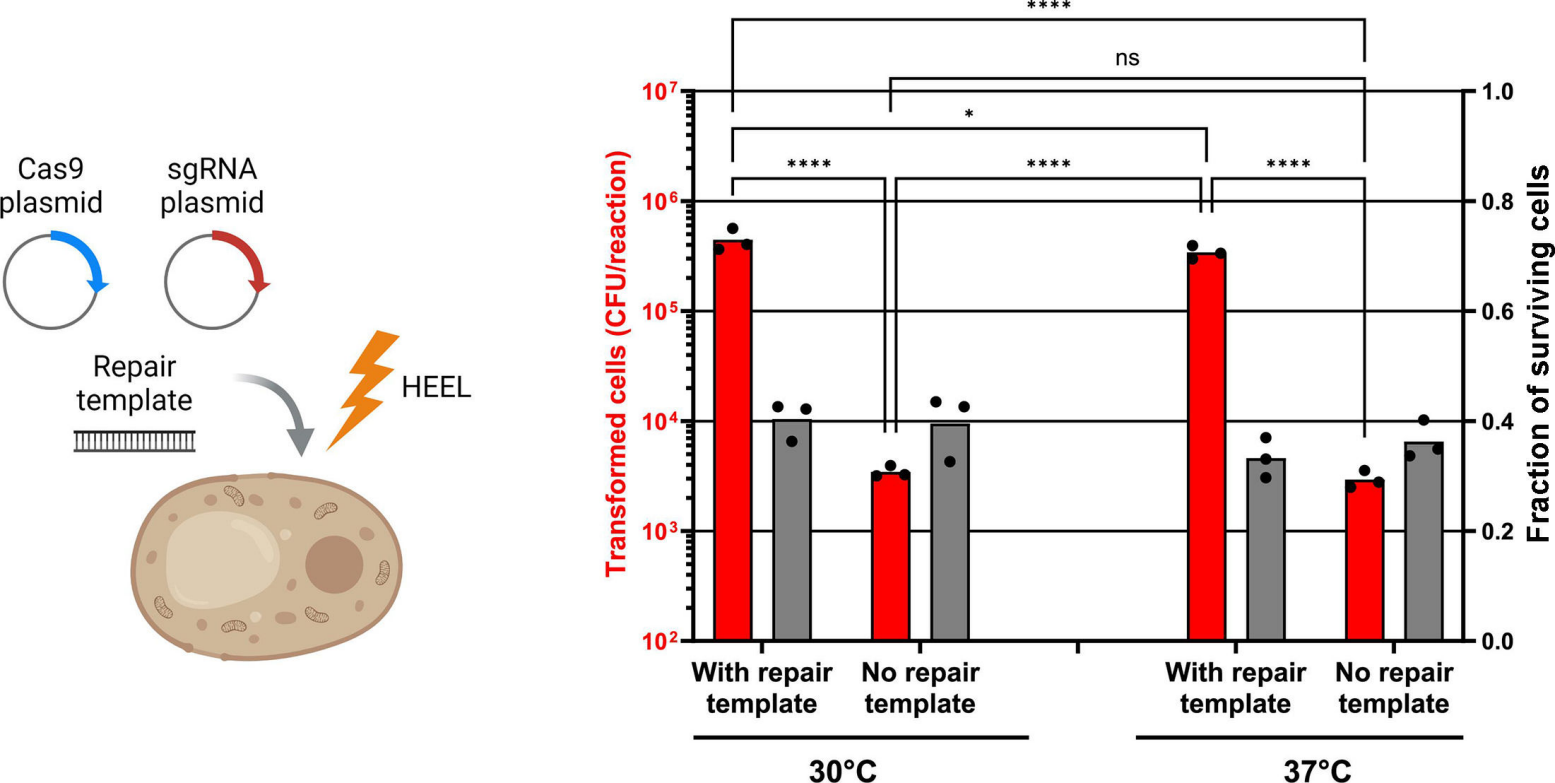
HEEL creates DNA libraries with near-perfect phenotype-to-genotype association that is almost 2-log larger than current high-throughput yeast genome integration methods. A *URA3* selection marker was integrated into the genome of *S. cerevisiae* strain CEN.PK2-1C using HEEL and the Cas9-aided EasyClone-MarkerFree genome integration strategy (24). Briefly, 100 µg of salmon sperm ssDNA and 4 µg of a FD XbaI (Thermo Fisher Scientific) linearized plasmid containing a *URA3* selection marker, 4.7 µg of a sgRNA plasmid (Leu^+^), and 6.3 µg of a Cas9 plasmid (His^+^) (1:1:1 mole ratio) was transformed into CEN.PK2-1C using the HEEL DNA transformation method (*n* = 3). Yeast cells were conditioned in 100 mM lithium acetate and 10 mM DTT at 30°C or 37°C for 60 min, then 6–9 × 10^8^ yeast cells per reaction were electroporated at 2.0 kV in a buffer containing 1 M sorbitol and 1 mM CaCl_2_. Transformed cells were enumerated on SC–His–Leu agar plates to select for both the sgRNA and Cas9 plasmids. Illustration created with BioRender.com. Statistical significance was calculated by two-way ANOVA with ns: *P* > 0.05, *: *P* ≤ 0.05, **: *P* ≤ 0.005, ***: *P* ≤ 0.0005, and ****: *P* ≤ 0.0001.

Genome integrations of DNA libraries in yeast is currently considered to be the gold standard for achieving high-quality libraries as each cell is transformed with one unique genotype. However, genome integrations in yeast via homologous recombination are significantly less efficient than plasmid transformations (23, 24). This is probably because the bottleneck during a genome integration is not only the amount of DNA transformed into each cell, but also the efficiency of the recombination event. Thus, when ultra-high-diversity libraries are needed, plasmid electroporation remains the most suitable method. To support this argument, we used HEEL together with the highly-efficient Cas9-aided genome integration method, EasyClone-MarkerFree, to integrate a *URA3* selection marker in the genome of our CEN.PK2-1C yeast strain (Fig. 7) (24). This *URA3* marker was designed to share approximately 500 bp of homology with the genome both upstream and downstream of the Cas9 cut site, which facilitates a highly efficient homologous recombination event. Using the HEEL method, we could achieve a genome integration yield of >10^5^ yeast cells per reaction (Fig. 7). This is almost a 2-log lower transformation yield compared with circular plasmid transformations, and almost a 3-log lower yield compared with linear plasmid transformations (Fig. 3B and C). Moreover, as EasyClone-MarkerFree requires that three separate DNA molecules are present in each yeast cell, i.e*.*, a Cas9 plasmid, a single-guide RNA (sgRNA) plasmid, and a linear repair template, we were also curious to investigate if a mild-heat shock would limit the efficiency of the *URA3* genome integration by limiting multi-plasmid uptake. Pre-treating yeast cells with a 37°C heat treatment before electroporation resulted in a lower than twofold decrease in the number of yeast cells with a *URA3* selection marker integrated in the genome (Fig. 7). However, we previously observed that pre-treating yeast cells with a 37°C heat treatment still allowed for approximately 30% of all yeast cells to be transformed with multiple plasmid molecules, compared with 80% of all yeast cells when the heat treatment was omitted. Thus, a two- to threefold decrease of the genome integration yield would be entirely in line with our previous observations (Fig. 5C and D).

The current utility of genome integrations is undeniable as a perfect phenotype-to-genotype association can be created for all transformed cells, although the libraries that can be created are small compared with those that can be created when transforming yeast with plasmid DNA, either linear or circular (5, 23, 24). In contrast, current high-throughput plasmid DNA transformation methods allow for the creation of large libraries but with low quality, i.e*.*, >80% of all cells are transformed with multiple unique genotypes (5, 7). Consequently, circular plasmid DNA transformations using the HEEL methodology is now a superior method that allows for the creation of yeast libraries with both high diversity (>10^7^ transformed cells per reaction) and high quality (approximately 70% mono-transformed cells).

## DISCUSSION

Multiplicity of plasmid transformations in bacteria and yeast is a well-known problem but also a scarcely investigated area of research. To the best of our knowledge, no study has designed any mitigation strategies to address this issue, beyond merely lowering the concentration of DNA used during a transformation. Though, this is a highly counterproductive strategy, decreasing the concentration of DNA during a transformation also decreases the number of transformed cells (transformation yield), i.e., there is an inverse correlation between the fraction of mono-transformed cells and the transformation yield. Here, we have demonstrated an easy and straight-forward yeast transformation method that significantly increases the fraction of mono-transformed yeast cells within a population, without sacrificing the transformation yield. Our strategy relies on exposing yeast cells to a mild 37°C heat-shock before electroporating the cells with both a circular plasmid and single-stranded salmon sperm DNA. Using this strategy (HEEL) we can increase the fraction of mono-transformed cells from 20% to 70%, while still maintaining a high transformation yield of >10^7^ transformed yeast cells per reaction. Thus, we can significantly increase the number of easy-to-screen high-quality yeast transformants with a clear phenotype-to-genotype relationship, while still retaining a high-diversity library. Current methods are restricted to generating either high-diversity libraries with low-resolution (multiple genotypes per cell), or high-resolution libraries, but with low diversity (few transformed cells) (5, 7, 23, 24). We expect that the HEEL methodology will simplify DNA, RNA, and/or protein library screenings in yeast to such an extent that high-diversity and high-resolution libraries can be generated, concurrently, with ease.

To the best of our knowledge, no study has reported that heat-shocking yeast cells before electroporation can elicit a beneficial effect on DNA transformations. Presumably this is because most studies have only investigated the effect that a traditional heat-shock temperature (42°C) has on yeast cells. And, as we show here, such high temperatures only have a detrimental effect on yeast plasmid uptake during an electroporation. Also, since a mild heat-shock temperature of 37°C does not appear to affect transformation yields in any significant way, it is easy to understand why this effect might have been overlooked by others. Although, as we show here, a mild heat-shock before electroporation does increase the fraction of transformed yeast cells, indicating that the efficiency of DNA uptake is increased simultaneously as more cells die by the heat-shock. Looking towards previously presented yeast DNA transformation models, it is possible that a novel synergistic effect might be happening, i.e*.*, that a membrane permeability effect, mediated by the electroporation, is occurring simultaneously with an endocytosis effect, mediated by lithium acetate, heat, and ssDNA. However, this is of course merely speculation, and we have intentionally chosen not to attempt to decipher the exact molecular mechanism behind this effect in this work. Moreover, our results clearly support previous findings that lithium acetate, DTT, ssDNA and elevated temperatures are all factors of paramount importance for a successful yeast DNA transformation (2, 3, 5, 16). However, we observe that these four parameters can, under some conditions, give rise to novel and unique transformation effects when applied in a nonconventional way, i.e*.*, by using heat-shock and electroporation simultaneously.

Circular plasmid DNA has previously been shown to transform yeast inefficiently (11). However, our findings presented here clearly demonstrate that by modifying the method described by Benatuil et al., by lowering the electroporation voltage from 2.5 to 2.0 kV and by adding 100 µg of single-stranded salmon sperm DNA to each transformation reaction, we can overcome this possible DNA topology issue and increase circular plasmid DNA transformation yields by almost 2-log (Fig. 1A and B, 3B and C). No other modifications were necessary to successfully transform yeast cells with a circular DNA molecule and achieve a high-throughput transformation yield that is comparable to other studies that have transformed yeast with linear DNA molecules (12–14). Consequently, with these innovations, we have made it possible to easily transform yeast cells with high-diversity libraries that first have been cloned and transformed into *E. coli*. This overcomes a major bottleneck for most yeast library transformations, as preparing enough high-diversity library DNA to transform yeast is not a trivial matter. The creation of high-diversity libraries in yeast requires >5 µg of DNA, while only <50 ng is required for an efficient DNA transformation of *E. coli* (1, 4, 6). By using the HEEL method described here, it is possible to first transform a high-diversity library into *E. coli*, allow the bacteria to amplify the plasmid DNA library, then extract the plasmid DNA and immediately transform this unmodified circular plasmid DNA library into yeast. Here, we completely omit the need to convert the plasmid to a linear molecule with homology arms to achieve high-efficiency transformations.

The development of NGS techniques has revolutionized the quantification and sequencing of high-diversity mutant libraries. However, the inherent weakness of any high-throughput NGS technique lies in its inability to link a single-cell phenotype to a specific genotype. A diverse library of mutants is often first screened for novel function(s), then each cell that exhibits a desired phenotype is selected/sorted and then pooled/binned together with all other cells with a similar phenotype. These mutants are then sequenced as a group to identify each individual genotype found in this sorted library. While this strategy generates a large pool of sequence data, it does not allow for a precise phenotype-to-genotype determination, merely an approximate phenotype-to-genotype relationship, as the range of different cellular phenotypes in each sorted library can differ by as much as one order of magnitude. In the past, this was usually not a source of concern, as most mutant-screening endeavors were designed to merely identify all beneficial mutants, of which a few clones were then chosen for further screening and characterization. However, with the introduction of AI and machine learning algorithms in most scientific fields, this is no longer the case. Predictive algorithms require precise phenotype-to-genotype measurements to generate insightful models with high accuracy. Approximate phenotype-to-genotype measurements are non-optimal as an AI training set. To create better AI-generated models, we need to strive to generate better data with better resolution. As shown here, the HEEL methodology can minimize the problem of phenotypic noise, i.e*.*, having multiple genotypes present in each yeast cell during a mutant screening. Moreover, our automated genotyping workflow allows for hundreds of yeast cells to be efficiently genotyped, which significantly simplifies the identification of beneficial mutants when using yeast as a screening platform (9). In addition, while the number of mutants screened and genotyped would be limited to just a few hundred, predictive algorithms should nevertheless be able to generate more insightful models from these smaller but more high-resolution data. This new approach should avoid the issues of binning sorted cells based on phenotype and then using the average phenotype of the group to perform a linear regression, which can lead to incorrect conclusions and mathematical models, as intrinsic noise in the data is overlooked (25).

## MATERIALS AND METHODS

### Strains, DNA constructs, and growth conditions

The bacterial and yeast strains, plasmid library, and oligos used in this study are listed in Tables S1 to S4. A detailed description of the dual-barcode plasmid library construction is available in the supplementary material and methods section of the appendix found in the online version of this article. Bacterial strains were routinely grown at 37°C and with shaking at 200 rpm in lysogeny broth (LB): 10 g/L tryptone, 5 g/L yeast extract, and 10 g/L NaCl. Media were supplemented with ampicillin at 100 mg/L or chloramphenicol at 25 mg/L when appropriate. Yeast strains were routinely grown at 30°C and with shaking at 200 rpm in yeast peptone dextrose broth (YPD): 20 g/L peptone, 10 g/L yeast extract, and 20  g/L glucose, or in synthetic drop-out medium, minus leucine (SC-Leu) (Sigma-Aldrich, #Y1376) or minus histidine (SC-His) (Sigma-Aldrich, #Y1751).

### *E. coli* DNA electroporation protocol

One hundred milliliters of a *E. coli* culture was grown to OD600 = 0.4 then centrifuged at 3,000×*g* for 10 min. The resulting pellet was pooled and re-suspended in 50 mL ultra-pure Milli-Q water. This procedure was repeated two more times, and the triple-washed cell pellet was then resuspended in a total volume of 400 µL Milli-Q water to achieve a final cell concentration of approximately OD600 = 100. Then, 50 µL of this concentrated cell solution and 1, 10, or 100 ng of plasmid DNA was transferred to a 1 mm MicroPulser electroporation cuvette (Bio-Rad), and the cells were electroporated at 1.8 kV, 25 µFD, and 200 Ohm, using a Gene Pulser Xcell Electroporation System (Bio-Rad). Subsequently, 500 µL of LB was added, and the cells were left to recover for 90 min at 37°C before being inoculated into LB-both or onto LB-agar with appropriate antibiotic selection. The number of viable cells before and after electroporation was determined by serial diluting each cell suspension and then plating all dilutions on non-selective LB agar plates. The number of transformed bacterial cells was enumerated by serial diluting each cell suspension and then plating all dilutions on selective LB-ampicillin agar plates.

### Heat-shock-electroporation (HEEL) yeast DNA transformation protocol

A detailed description of the HEEL yeast DNA transformation workflow can be found in the supplemental material. For the preliminary HEEL method optimization (Fig. 2A through F; Fig. S1A through F), the transformation protocol was carried out exactly as the optimized HEEL protocol (see supplemental material) except that yeast cells were conditioned in a solution of 100 mM lithium acetate and 20 mM dithiothreitol (DTT) at either 30°C or 42°C for 1 h. Cells were then electroporated with 5 µg of plasmids DNA and 100 µg of salmon sperm ssDNA, unless otherwise stated. The number of viable cells before and after electroporation was determined by serial diluting each cell suspension and then plating all dilutions on non-selective YPD agar plates. The number of transformed yeast cells was enumerated the same way, but cells were plated on selective SC-His or SC-Leu agar plates.

### Cas9 and heat-shock-electroporation (HEEL) mediated genome integration protocol

The genome integration protocol was carried out exactly as the optimized HEEL protocol (see supplemental material) except that yeast cells were electroporated with 100 µg of salmon sperm ssDNA and 4 µg of a FD XbaI (Thermo Fisher Scientific) linearized plasmid containing the *URA3* selection marker, 4.7 µg of the sqRNA plasmid and 6.3 µg of the Cas9 plasmid (Table S3), following the EasyClone-MarkerFree genome integration protocol (24). This amount of DNA created a 1:1:1 mole ratio of all three DNA molecules, excluding the ssDNA, while simultaneously limiting the total amount of DNA, excluding the ssDNA, to 15 µg. The number of viable cells before and after electroporation was determined by serial diluting each cell suspension and then plating all dilutions on non-selective YPD agar plates. The number of transformed yeast cells was enumerated the same way, but the cells were plated on double-selective SC-His-Leu agar plates. This selected for both the sgRNA and Cas9 plasmids, thus ensuring that only cells that modified the Cas9 cut site *via* homologous recombination of the *URA3* marker would survive the Cas9-mediated DNA cleavage.

### Automated yeast genotyping workflow

A detailed description of the automated yeast genotyping workflow can be found in the supplemental material.

### MiSeq NGS sequencing

Yeast single-cell-derived colony PCR-amplified amplicons were pooled according to experimental conditions, purified using a silica DNA-binding column, and then sequenced with a MiSeq Reagent Micro Kit v2 (300 Cycles) on an Illumina MiSeq instrument, according to manufacturer’s instructions. The pooled libraries were sequenced at a concentration of 10 pM, with 12.5 pM PhiX Control v3 (Illumina) added. A custom read primer (oligo 358) and a custom index primer (oligo 359) were used at a final concentration of 0.5 µM during the MiSeq sequencing to overcome potential issues with primer–dimer fragment sequencing (Table S4).

### NGS analysis

A detailed description of the Illumina NGS sequencing analysis can be found in the supplemental material.

## SUPPLEMENTAL MATERIAL

Supplemental Information mbio.03197-24-s0001.pdf Additional experimental details and supplemental figures and tables.

ASM does not own the copyrights to Supplemental Material that may be linked to, or accessed through, an article. The authors have granted ASM a non-exclusive, world-wide license to publish the Supplemental Material files. Please contact the corresponding author directly for reuse.
