# Supplementary material for: Accurate phenotype-to-genotype mapping of high-diversity yeast libraries by heat-shock-electroporation (HEEL)
Source: mBio. 2024 Dec 20;16(2):e03197-24. doi: 10.1128/mbio.03197-24 (PMC11796364; doi:10.1128/mbio.03197-24)
Supplement: Supplemental Information — Additional experimental details and supplemental figures and tables. [file mbio.03197-24-s0001.pdf]

Supplementary Information for:

Accurate phenotype-to-genotype mapping of high-diversity yeast libraries by heat-shock-electroporation (HEEL)

Marcus Wäneskog*[1], Emma Elise Hoch-Schneider[1], Shilpa Garg[1], Christian Kronborg Cantalapiedra[1], Elena Schaefer[2], Michael Krogh Jensen[1], Emil Damgaard Jensen*[1]

[1] The Novo Nordisk Foundation Center for Biosustainability, Technical University of Denmark, DK-2800 Kgs. Lyngby, Denmark

[2] Formerly: The Novo Nordisk Foundation Center for Biosustainability, Technical University of Denmark, DK-2800 Kgs. Lyngby, Denmark

* To whom correspondence should be addressed. Marcus Wäneskog, e-mail: marcusw@biosustain.dtu.dk or Emil D. Jensen, e-mail: emdaje@biosustain.dtu.dk

This supplementary file includes:

Supplementary methods

Supplementary tables S1-S4

Supplementary figures S1-S3

Yeast heat-shock-electroporation (HEEL) DNA transformation workflow

60 mL YPD broth (1% (w/v) yeast extract, 2% (w/v) meat-derived peptone and 1% (w/v) glucose) was inoculated with a fresh re-streaked yeast colony and incubated at 30 °C with vigorous aeration for 24h, or until the yeast culture reached stationary phase.

40 mL of the stationary phase yeast culture was then added to 400 mL of fresh YPD broth and incubated at 30 °C with vigorous aeration for approximately 3-4h, until reaching OD600 = 1.

400 mL of the OD600 = 1 yeast culture was collected and centrifuged at 3000xg for 5min. The YPD supernatant was discarded, and the resulting yeast cell pellet was collected and pooled in a total volume of 50 mL pre-chilled (4°C) ultrapure Milli-Q Water. This washing step was repeated one additional time.

The resulting yeast pellet was then resuspended in 50 mL of a 1 M sorbitol and 1 mM CaCl2 solution.

The osmotically shocked yeast cells were then centrifuged again at 3000xg for 5min, and the sorbitol solution was discarded, and the yeast cells were resuspended in 50 mL of a 100 mM Lithium Acetate (LiAc) and 10 mM Dithiothreitol (DTT) solution. The LiAc and DTT solution was then incubated at 37 °C with vigorous aeration for 1h.

The mildly heat-shocked cells were then pelleted at 3000xg for 5min and resuspended in 50 mL of a 1 M sorbitol and 1 mM CaCl2 solution. After an additional centrifugation at 3000xg for 5min, the pelleted yeast cell solution was re-suspended in a 1 M sorbitol and 1 mM CaCl2 solution, in a total volume of 4 mL.

400 µL of the yeast solution was used per transformation reaction by mixing the cell suspension with 5-10 µg of circular plasmid DNA (preferable at a concentration of 1-2 µg/µl) and 100 µg of boiled single-stranded salmon sperm DNA (ssDNA), at a concentration of 10 µg/µl. The plasmid DNA and ssDNA mixture was then transferred to a MicroPulser electroporation cuvette (Bio-Rad) with a 2 mm gap. The solution was immediately electroporated at 2.0 kV, 200 Ohm and 25 µFD, using a Gene Pulser Xcell Electroporation System (Bio-Rad). This resulted in a time constant of approximately 3.5-4.2 ms. Directly after the electroporation (within 5sec), 2 mL of fresh YPD was added to the electroporated cells and the solution was incubated at 30 °C with vigorous aeration for 1h to allow cells to recover.

Starting/input cells, surviving cells and transformed cells were all enumerated by first serial diluting the starting cell solution and the recovered cell suspension. Each dilution was inoculated on a YPD (surviving cells) and selective media (transformed cells) agar plate. Yeast colonies were enumerated after 48h of growth at 30 °C.

Automated yeast genotyping workflow

100 µL of a yeast cell wall digestion solution, containing 10 mM TRIS-HCl pH 8, 1 mM Ethylenediaminetetraacetic acid (EDTA), 10 mM Dithiothreitol (DTT) and 1000 U/mL Lyticase from Arthrobacter luteus (Sigma-Aldrich), were dispensed equally into a 96-well plate (Thermo Fisher Scientific) using an OT-2 liquid handling robot (Opentrons).

Individual single-cell derived yeast colonies were randomly picked using a Singer PIXL Precision Microbial Colony Picker (Singer Instruments) and inoculated into each of the pre-aliquoted 96 wells,

using the following settings:
Pinning Dry (Speed: 19 mm/s, Pickup Line Length: 10 mm)
Mixing Dry (Speed: 20 mm/s, Radius: 0.4 mm, Vertical Travel: 2.5 mm, Cycles: 1)
Pinning Wet (Speed: 60 mm/s, Well Base Clearance: 20% of well, Nozzle Clearance: 2 mm)
Mixing Wet (Speed: 60 mm/s, Radius: 50% of available, Revolutions: 3)

The Lyticase and yeast colony mixture were incubated at 37 °C for 1 hour. Then 90 µL of the mixture was removed and discarded by an OT-2 Robot (Opentrons). The remaining 10 µL was mixed with 14 µL of a sterile MilliQ H_2O solution containing a universal forward primer (oligo; 348).

9 separate RedTaq DNA polymerase master mixes (Sigma-Aldrich), one per experiment, was prepared and mixed with experiment specific reverse primers (oligos; 349-357). 26 µL of each PCR master mix was added to each of the yeast cell solutions, using an OT-2 Robot (Opentrons). The 96-well plate was then incubated in a T100 Thermocycler (Bio-Rad), using the following settings:

1) 95°C for 10 min

2) 95°C for 25sec
3) 61°C for 30sec
4) 72°C for 30sec.

Step 2-4 was repeated for 35 cycles.

5) 72°C for 5min

Following the PCR amplification, 15 µL from 31 individual PCR reactions per experiment was transferred to a 96-well sequencing plate, leaving 3 empty wells as negative controls, and Sanger sequenced (Eurofins Genomics) using oligo; 288. The remaining 35 µL of each PCR reaction was purified and pooled for Illumina MiSeq NGS and analysis.

MiSeq NGS analysis workflow

The NGS analysis was performed in Python using the following libraries: Biopython, matplotlib, Collections, Pandas. The following steps outline the data processing:

Sequence Filtering by Phred score:
NGS fastq files were filtered based on quality using the LUMC fastq filter (https://github.com/LUMC/fastq-filter, last accessed September 22nd, 2024). Sequences with a quality score less than Q=35 (a calculated error rate of 0.0003162 given that $Q = -10\log_{10}e$, with e as the error rate) were filtered and discarded. This ensured that only high-quality sequences were retained for further analysis.

Alignment to Reference Sequence:
The filtered sequences were aligned to a reference sequence (TGCGCCAGCTTTCATCCCCGATATGCACCACCGGGTAAAGTTCACCATAGACTCATAANNNNNNNNNNTTATGAGACTTTATCTGACAGCAGACGTGCACTGGCCAGGGGGATCACCATCCGTCGCCCGGG) using a global pairwise alignment with a gap penalty of -1. This alignment treated 'N' as either A, C, T, or G and sequences were then trimmed to a predefined region for uniform length (position 36 to 91 of the reference sequence).

Sequence Counting:
After the reference sequence alignment, all unique sequences were identified and counted in each of the 9 experimental libraries. Sequences with a coverage of more than 200-fold were retained for further analysis.

Similarity analysis
A custom Python script was used to identify all barcode sequences that were identical, or only differed by a single nucleotide, across libraries. This script compares all sequences across all libraries. If an identical sequence was found in another library, it was recorded as “identical”, and no further comparisons were made for that sequence. Similarly, if a sequence was found to differ by only one nucleotide from any other sequence in any library, it was counted as a “near-identical”, and the comparison process for that sequence stopped. This approach ensured that each sequence was only counted once per library for either identical or near-identical sequence differences.

Visualization and Reporting:
The processed data, including the counts of unique sequences and nucleotide frequencies, was reported and visualized with matplotlib. For plotting of nucleotide frequencies, all sequences with indels, compared to the reference sequence, were excluded to more accurately visualize the bias within the dual barcode region.

Construction of the pRS413-CEN/ARS-ccdB-cat plasmid

The 2-micron origin of replication of the previously described pESC-HIS-ccdB-USER (pCfB55) plasmid was changed by yeast *in vivo* recombination by first cutting the 2µ origin of replication with FD XbaI (Thermo Fisher Scientific), thus linearizing the plasmid (1). The previously described pRS413 plasmid, which has a CEN/ARS origin of replication, but otherwise shares an identical backbone to the pCfB55 plasmid, was linearized by cutting-out a fragment containing the CEN/ARS origin of replication flanked by partial AmpR and HIS3 CDS sequences, using FD HindIII (Thermo Fisher Scientific) and BsrDI (New England Biolabs) (2). The pRS413 fragment containing the CEN/ARS origin of replication and the linearized pCfB55 plasmid was co-transformed into CEN.PK110-10C (MW2) using the lithium acetate ssDNA heat-shock method to allow for homologues recombination of the two fragments (3). Yeast colonies that were histidine prototrophs were PCR screened using oligos; 73 and 17031, to identify positive recombination events. The recombined yeast plasmid (pMW1) was extracted using the Zymoprep Yeast Plasmid Miniprep Kit (Zymo Research) and transformed into a CcdB resistant *E. coli* strain (MW69) (Thermo Fisher Scientific). Following a plasmid extraction from *E. coli*, the entire plasmid was verified by whole plasmid sequencing using Oxford Nanopore Technologies (ONT).

Construction of the Dual-Barcoded pUC-ccdB-cat plasmid

The pUC19 backbone along with the chloramphenicol resistance marker and the CcdB counterselection marker was PCR amplified with oligos; 141 and 142, from the pRS413-CEN/ARS-ccdB-cat plasmid (pMW1). The resulting linear DNA was self-circularized, using T4 DNA ligase (Thermo Fisher Scientific), and then transformed into a CcdB resistant *E. coli* strain (MW69) (Thermo Fisher Scientific). Plasmids from several chloramphenicol resistant bacterial colonies were extracted and verified by sequencing, using oligo; 237. This new pUC19-CatR-CcdB vector (pMW17) was then used as template for a PCR reaction using a mix of 12 individual oligos (272-283). Introducing the dual-barcoded sequences into the middle of the ccdB CDS. The resulting linear DNA was self-circularized at a final concentration of 1 ng/µL, using T4 DNA ligase (Thermo Fisher

Scientific). This circular molecule was transformed via electroporation into the CcdB sensitive *E. coli* strain TOP10 (MW150) (Thermo Fisher Scientific), selecting for plasmids with a ccdB gene that was disrupted by an insertion of the dual-barcoded sequence. Plasmid selection and propagation post-transformation was done entirely in liquid culture to ensure maximum diversity. The entire plasmid library (pMW35) was extracted and sequenced with oligo; 288, to verify diversity and the presence of the dual-barcode.

Construction of the Dual-Barcoded pRS413-CEN/ARS-ccdB-cat plasmid

The pRS413-CEN/ARS-ccdB-cat plasmid (pMW1) was linearized by cutting in the middle of the ccdB CDS with FD SmaI (Thermo Fisher Scientific). This linear pRS413-CEN/ARS-ccdB-cat fragment was then co-transformed into CEN.PK110-10C (MW2) along with the Dual-Barcoded pUC-ccdB-cat plasmid library (pMW35), using the HEEL yeast DNA transformation methodology. This allowed the Dual-Barcoded pUC-ccdB-cat library to be used as a gap-repair template for the linear pRS413-CEN/ARS-ccdB-cat fragment, ensuring a recombination of the dual-barcoded region from the pUC-ccdB-cat plasmids to the pRS413-CEN/ARS-ccdB-cat plasmid. The resulting yeast plasmid library (MW165) was then extracted using the Zymoprep Yeast Plasmid Miniprep Kit (Zymo Research) and transformed into the CcdB sensitive *E. coli* strain TOP10 (MW150) (Thermo Fisher Scientific). Selecting for pRS413-CEN/ARS-ccdB-cat plasmids with a disrupted ccdB gene. The resulting counter-selected Dual-Barcoded yeast plasmid library was then amplified in TOP10 *E. coli* cells (pMW39) before being extracted and sequenced with oligo; 288, to verify sequence diversity and the presence of the dual-barcode region.

Preparation of linear vector and insert DNA for yeast gap-repair transformation

The pYB-Dual vector (pMW22) was linearized by FD XhoI and FD BamHI (Thermo Fisher Scientific) and dephosphorylated by FastAP (Thermo Fisher Scientific), thus creating a linear plasmid molecule with incompatible sticky-ends. This linearized plasmid DNA was purified by DNA binding column. The *HIS3* marker from the pRS413 plasmid (pMW1) was PCR-amplified with oligos; 372 and 373, adding 40bp long homology-arms corresponding to the ends of the FD XhoI and FD BamHI (Thermo Fisher Scientific) cleaved pYB-Dual vector. This PCR-amplified DNA was purified by DNA binding column.

Preparation of linear repair template for yeast genome integration

The *URA3* repair template from plasmid pCfB385 was cleaved-out by FD XbaI (Thermo Fisher Scientific) and used together with the sgRNA plasmid; pCfB3045, and the Cas9 plasmid; pCfB5270, to facilitate a high-efficiency genome integration of the *URA3* marker, following the EasyClone-MarkerFree strategy (4). The cleaved repair template containing plasmid (pCfB385) was purified by DNA binding column.

Table S1. Bacterial strains used in this study.

Strain number	Genotype	Origin
MW69	*E. coli* One Shot ccdB Survival 2 T1R *F- mcrA Δ(mrr-hsdRMS-mcrBC) φ80lacZΔM15 ΔlacX74 recA1 araΔ139 Δ(ara-leu)7697 galU galK rpsL(StrR) endA1 nupG fhuA::IS2*	Thermo Fisher Scientific
MW150	*E. coli* TOP10 *F- mcrA Δ(mrr-hsdRMS-mcrBC) φ80lacZΔM15 ΔlacX74 recA1 araD139 Δ(ara-leu)7697 galU galK λ–rpsL(StrR) endA1 nupG*	Thermo Fisher Scientific

Table S2. Yeast strains used in this study.

Strain number	Genotype	Origin
MW2	CEN.PK110-10C *MATα his3Δ1*	Lab Collection
MW23	CEN.PK2-1C *MATa ura3-52 his3Δ1 leu2-3,112 trp1-289 MAL2-8c SUC2*	Lab Collection
MW71	BY4741 *MATa his3Δ1 leu2Δ0 met15Δ0 ura3Δ0*	Lab Collection
MW165	CEN.PK110-10C *MATα his3Δ1* /pRS413(CEN6-ARS4 ccdB::(low/high-complexity-barcode-library)-cat) His+ AmpR CatR ***(library)***	This Study
MW168	EBY100 *MATa aga1::pGAL1-aga1-URA3 ura3-52 trp1 leu2-Δ200 his3-Δ200 pep4::HIS3 prbd1.6R can1 GAL*	Lab Collection

Table S3. Plasmids used in this study.

Plasmid name	Genotype	Origin
pCfB55	pESC-HIS-ccdB-USER	(1)
pCfB385	pXI-3-site-USER-URA3 Ura+	(5)
pCfB3045	psgRNA(XI-3-site) Leu+	(4)
pCfB5270	pCas9 His+	(4)
pMW1	pRS413(CEN6-ARS4 ccdB-cat) His+ AmpR CatR	This Study
pMW17	pUC19(ccdB-cat) CatR	This Study
pMW22	pYB-Dual(Empty) Leu+ AmpR	(6)
pMW35	pUC19(ccdB::(low/high-complexity-barcode)-cat) CatR ***(library)***	This Study
pMW39	pRS413(CEN6-ARS4 ccdB::(low/high-complexity-barcode)-cat) His+ AmpR CatR ***(library)***	This Study
pRS413	pUC19 CEN6-ARS4 AmpR His+	(2)

Table S4. Oligos used in this study.

Name	Sequence 5'-3'
73	GGAACAACACTCAACCCTATCTCG
141	5'Phosphate-CCTTTTTGATAATCTCATGACC
142	GCAGCCTACTCGCTATTGTC
237	CTCTTTTGCTGACGAGAACAGG
272	5'Phosphate-GTGAACTTTACCCGGTGGTGC
273	CATAHACTCATAANNNNNNNNNNNTTATGAGACTTTATCTGACAGCAGACG
274	CATAGBCTCATAANNNNNNNNNNNTTATGAGACTTTATCTGACAGCAGACG

275	CATAGADTCATAANNNNNNNNNNNTTATGAGACTTTATCTGACAGCAGACG
276	CATAGACVCATAANNNNNNNNNNNTTATGAGACTTTATCTGACAGCAGACG
277	CATAGACTDATAANNNNNNNNNNNTTATGAGACTTTATCTGACAGCAGACG
278	CATAGACTCBTAANNNNNNNNNNNTTATGAGACTTTATCTGACAGCAGACG
279	CBTAGACTCATAANNNNNNNNNNNTTATGAGACTTTATCTGACAGCAGACG
280	CAVAGACTCATAANNNNNNNNNNNTTATGAGACTTTATCTGACAGCAGACG
281	CATBGACTCATAANNNNNNNNNNNTTATGAGACTTTATCTGACAGCAGACG
282	CATAGACTCAVAANNNNNNNNNNNTTATGAGACTTTATCTGACAGCAGACG
283	CATAGACTCATBANNNNNNNNNNNTTATGAGACTTTATCTGACAGCAGACG
288	TCGCGGTGGCTGAGATCAGC
348	AATGATACGGCGACCACCGAGATCTACACTAGATCGCTCGTCGGCAGCGTCCATGC GCCAGCTTTCATCC
349	CAAGCAGAAGACGGCATACGAGATCGCTCAGTTCGTCTCGTGGGCTCGGCAGAGT GATATTATTGACACG
350	CAAGCAGAAGACGGCATACGAGATTATCTGACCTGTCTCGTGGGCTCGGCAGAGT GATATTATTGACACG
351	CAAGCAGAAGACGGCATACGAGATATATGAGACGGTCTCGTGGGCTCGGCAGAGT GATATTATTGACACG
352	CAAGCAGAAGACGGCATACGAGATCTTATGGAATGTCTCGTGGGCTCGGCAGAGT GATATTATTGACACG
353	CAAGCAGAAGACGGCATACGAGATTAATCTCGTCGTCTCGTGGGCTCGGCAGAGT GATATTATTGACACG
354	CAAGCAGAAGACGGCATACGAGATGCGCGATGTTGTCTCGTGGGCTCGGCAGAGT GATATTATTGACACG
355	CAAGCAGAAGACGGCATACGAGATAGAGCACTAGGTCTCGTGGGCTCGGCAGAGT GATATTATTGACACG
356	CAAGCAGAAGACGGCATACGAGATTGCCTTGATCGTCTCGTGGGCTCGGCAGAGT GATATTATTGACACG
357	CAAGCAGAAGACGGCATACGAGATCTACTCAGTCGTCTCGTGGGCTCGGCAGAGT GATATTATTGACACG
358	GATAACGGAGACCGGCACACTGGCCATATCG
359	CGTGTCAATAATATCACTCTGCCGAGCCCACGAGAC
372	ACTCACTATAGGGCGAATTGGGTACCGGGCCCCCCCTCGAACCTCTGACACATGCA GCTCC
373	TTCCTTTTCGGTTAGAGCGGATGAATGCACGCGATGGATCCAACACTCAACCCTAT CTCGGTC
17031	AGGGGTUCCGCGCACATTTCCCCGAAAAGT

Fig. S1

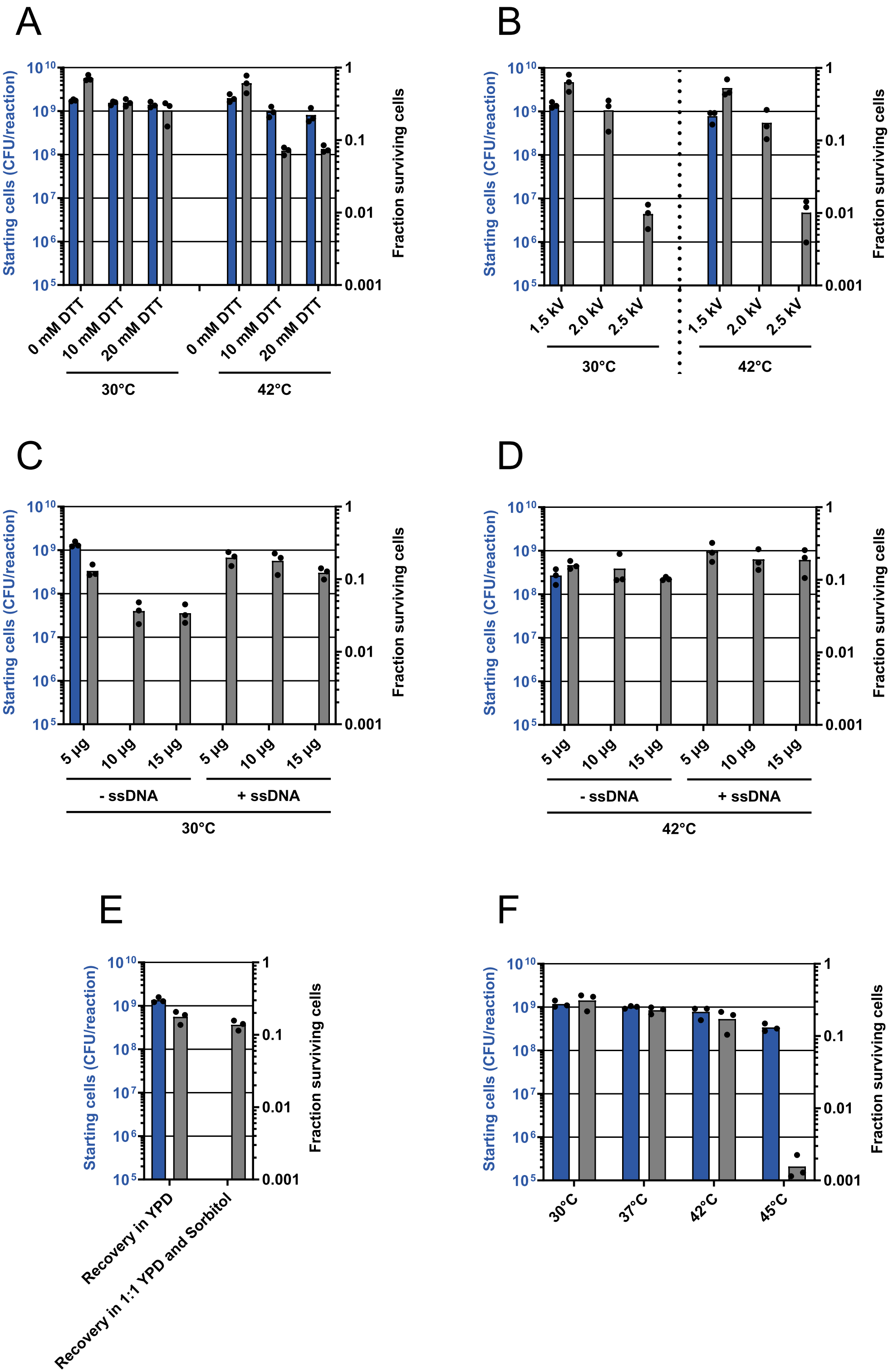

A
Starting cells (CFU/reaction)
Fraction surviving cells
0 mM DTT
10 mM DTT
20 mM DTT
30°C
42°C
B
1.5 kV
2.0 kV
2.5 kV
30°C
42°C
C
5 µg
10 µg
15 µg
- ssDNA
+ ssDNA
30°C
D
5 µg
10 µg
15 µg
- ssDNA
+ ssDNA
42°C
E
Recovery in YPD
Recovery in 1:1 YPD and Sorbitol
F
30°C
37°C
42°C
45°C

Figure S1.

The resulting change in yeast cell survivability post-electroporation when modifying each parameter of the HEEL DNA transformation method. *S. cerevisiae* strain CEN.PK110-10C was transformed with 5 µg of a pRS413 (His^+) circular plasmid molecule and 100 µg of salmon sperm single-strand DNA (ssDNA), using a preliminary HEEL DNA transformation workflow. Briefly, yeast cells were conditioned in 100 mM lithium acetate and 20 mM DTT at 30 °C or 42 °C for 60min, then electroporated at 2.0 kV in a buffer containing 1 M sorbitol and 1 mM $CaCl_2$, unless otherwise stated. A) The effect on yeast survivability by changing the DTT concentration, B) the electroporation voltage, C-D) plasmid DNA concentration and adding ssDNA, E) omitting sorbitol from the recovery media, or F) changing the heat-shock temperature (n=3). The enumeration of viable starting cells was made after conditioning cells in lithium acetate and DTT, but before electroporation. Thus, when the yeast cell conditioning was harsh the number of viable cells for electroporation was reduced. When only the electroporation or recovery parameter was changed, the same solution of conditioned starting cells (blue bars) was used for multiple investigations.

Fig. S2

A

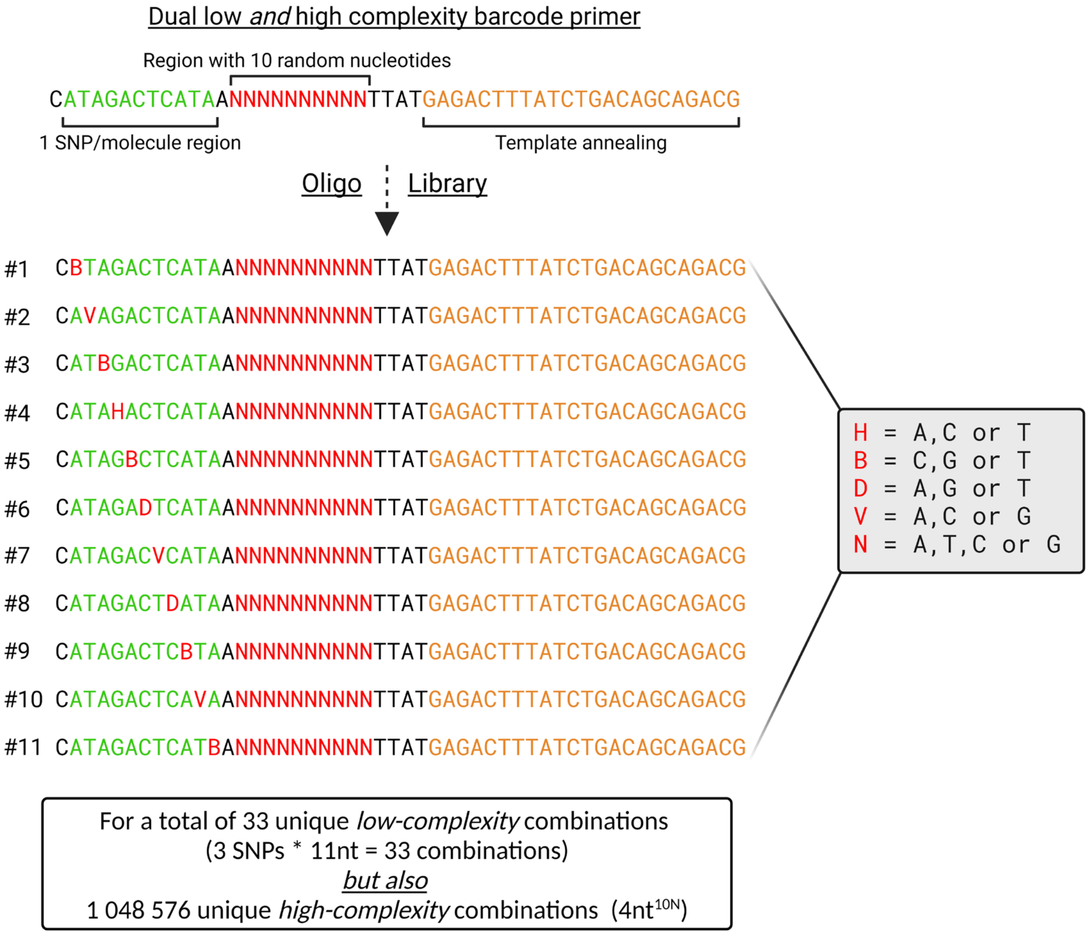

B

33 SNPs Barcode	Plasmids/cell					
	1 Plasmid	2 Plasmids	3 Plasmids	4 Plasmids	5 Plasmids	6 Plasmids
Probability that all plasmids have unique SNPs	1.00	0.97	0.91	0.83	0.73	0.62
Probability of at least one SNP recurring more than once	0.00	0.03	0.09	0.17	0.27	0.38

Example calculations for 33 SNPs barcode:
Probability that 1 plasmid is unique = (33barcodes/33barcodes)
Probability that 2 plasmids are both unique = (32barcodes-left/33barcodes) x (33barcodes/33barcodes)
Probability that 3 plasmids are all unique = (31barcodes-left/33barcodes) x (32barcodes-left/33barcodes) x (33barcodes/33barcodes)

10N Barcode	Number of random nucleotides									
	1	2	3	4	5	6	7	8	9	10
	Probability that at least one position contains one unique nucleotide for every unique plasmid transformed									
1 Plasmid/cell	1.00	1.00	1.00	1.00	1.00	1.00	1.00	1.00	1.00	1.00
2 Plasmids/cell	0.75	0.94	0.98	1.00	1.00	1.00	1.00	1.00	1.00	1.00
3 Plasmids/cell	0.38	0.61	0.76	0.85	0.90	0.94	0.96	0.98	0.99	0.99
4 Plasmids/cell	0.09	0.18	0.26	0.33	0.39	0.45	0.50	0.55	0.59	0.63

Example calculations for 10N barcode:
Probability that one random nucleotide (A, T, C or G) is unique for every plasmid when 1 plasmid is transformed = (4nt/4nt)
Probability that one random nucleotide (A, T, C or G) is unique for every plasmid when 2 plasmids are transformed = (3nt-left/4nt) x (4nt/4nt)
Probability that one random nucleotide (A, T, C or G) is unique for every plasmid when 3 plasmids are transformed = (2nt-left/4nt) x (3nt-left/4nt) x (4nt/4nt)

Probability that at least 1 of 5 random nucleotides are unique for every plasmid when 1 plasmid is transformed = 1-((1-(4nt/4nt))^5random-nt)
Probability that at least 1 of 5 random nucleotides are unique for every plasmid when 2 plasmids are transformed = 1-((1-((3nt-left/4nt) x (4nt/4nt)))^5random-nt)
Probability that at least 1 of 5 random nucleotides are unique for every plasmid when 3 plasmids are transformed = 1-((1-(((2nt-left/4nt) x (3nt-left/4nt) x (4nt/4nt)))^5random-nt)

Figure S2.

The design of the dual-barcoded library. A) Schematic illustration of the 11 different oligo sequences required to create the dual-barcoded plasmid library. Illustration created with BioRender.com. B) Probabilities of correctly enumerating the number of plasmids per cell if using Sanger sequencing and either the SNP or high-diversity (10N) barcode approach.

Fig. S3

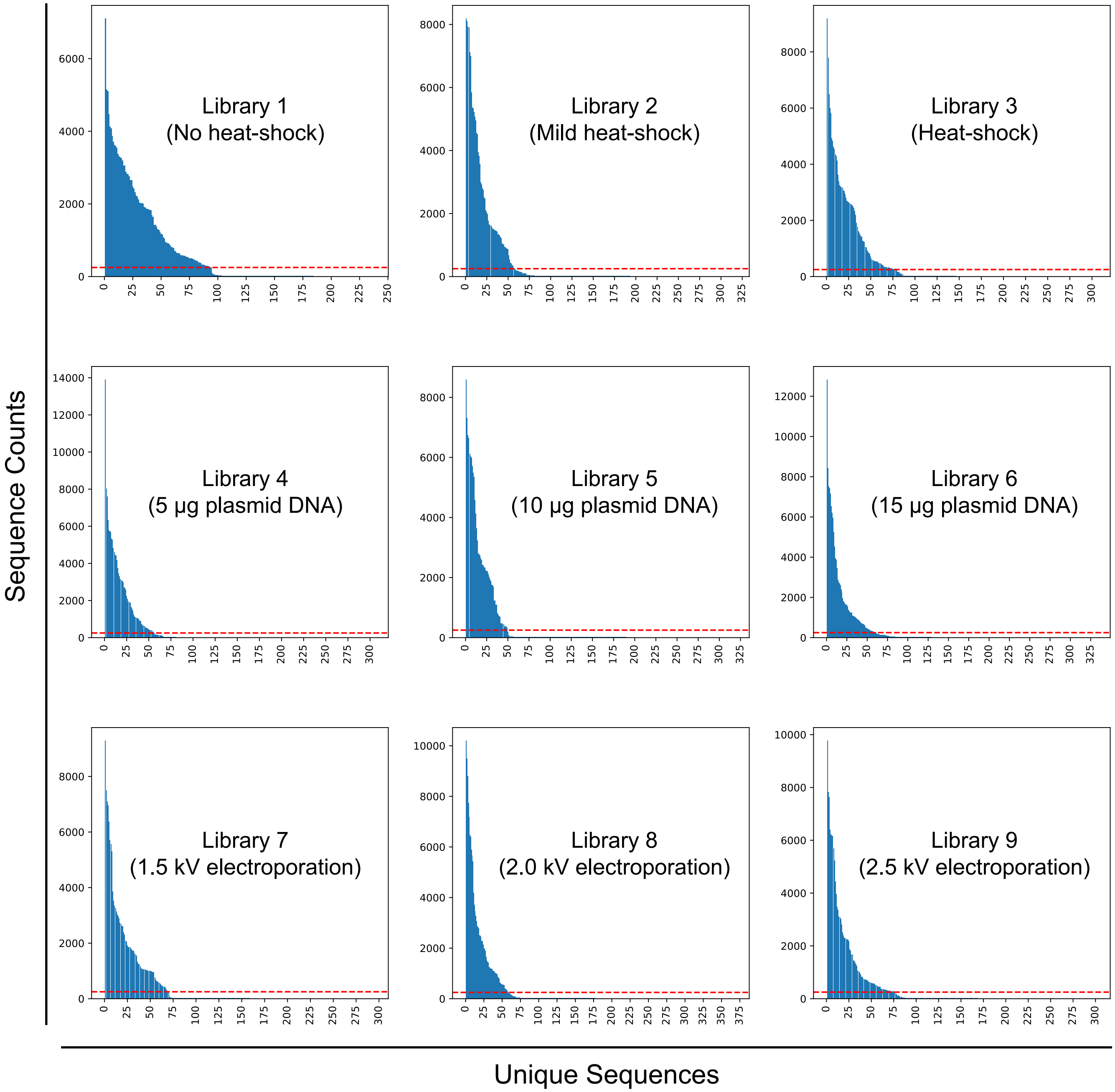

Sequence Counts
Unique Sequences
Library 1
(No heat-shock)
Library 2
(Mild heat-shock)
Library 3
(Heat-shock)
Library 4
(5 μg plasmid DNA)
Library 5
(10 μg plasmid DNA)
Library 6
(15 μg plasmid DNA)
Library 7
(1.5 kV electroporation)
Library 8
(2.0 kV electroporation)
Library 9
(2.5 kV electroporation)

Figure S3.

A 200-fold coverage best fits the distribution of the NGS data. The number of identified unique barcodes and the occurrence, i.e., coverage, of each sequence in all nine NGS libraries (experimental conditions). The dashed red lines represent a 200-fold coverage.

References

1. Jensen NB, Strucko T, Kildegaard KR, David F, Maury J, Mortensen UH, Forster J, Nielsen J, Borodina I. 2014. EasyClone: method for iterative chromosomal integration of multiple genes in Saccharomyces cerevisiae. FEMS Yeast Res 14:238–48.

2. Sikorski RS, Hieter P. 1989. A system of shuttle vectors and yeast host strains designed for efficient manipulation of DNA in Saccharomyces cerevisiae. Genetics 122:19–27.

3. Gietz RD, Schiestl RH. 2007. High-efficiency yeast transformation using the LiAc/SS carrier DNA/PEG method. Nat Protoc 2:31–34.

4. Jessop-Fabre MM, Jakočiūnas T, Stovicek V, Dai Z, Jensen MK, Keasling JD, Borodina I. 2016. EasyClone-MarkerFree: A vector toolkit for marker-less integration of genes into Saccharomyces cerevisiae via CRISPR-Cas9. Biotechnol J 11:1110–7.

5. Mikkelsen MD, Buron LD, Salomonsen B, Olsen CE, Hansen BG, Mortensen UH, Halkier BA. 2012. Microbial production of indolylglucosinolate through engineering of a multi-gene pathway in a versatile yeast expression platform. Metab Eng 14:104–111.

6. Wäneskog M, Rasmussen TB, Jensen ED. 2024. A strategy for successful dual-species protein expression of genes with non-optimal codon usage destined for bacterial and yeast cell factories. Biotechnol Prog https://doi.org/10.1002/btpr.3482.
